# Tandem Molecular Self-Assembly Selectively Inhibits Lung Cancer Cells by Inducing Endoplasmic Reticulum Stress

**DOI:** 10.34133/2019/4803624

**Published:** 2019-12-03

**Authors:** Debin Zheng, Yumiao Chen, Sifan Ai, Renshu Zhang, Zhengfeng Gao, Chunhui Liang, Li Cao, Yaoxia Chen, Zhangyong Hong, Yang Shi, Ling Wang, Xingyi Li, Zhimou Yang

**Affiliations:** ^1^Key Laboratory of Bioactive Materials, Ministry of Education, College of Life Sciences, Key Laboratory of Medicinal Chemical Biology, Collaborative Innovation Center of Chemical Science and Engineering, and National Institute of Functional Materials, Nankai University, Tianjin 300071, China; ^2^School of Ophthalmology & Optometry and Eye Hospital, Wenzhou Medical University, and Wenzhou Institute of Biomaterials and Engineering, CNITECH, CAS, Wenzhou 325035, China; ^3^College of Pharmacy, Nankai University, Tianjin 300071, China; ^4^Jiangsu Center for the Collaboration and Innovation of Cancer Biotherapy, Cancer Institute, Xuzhou Medical University, Xuzhou, Jiangsu, China

## Abstract

The selective formation of nanomaterials in cancer cells and tumors holds great promise for cancer diagnostics and therapy. Until now, most strategies rely on a single trigger to control the formation of nanomaterials *in situ*. The combination of two or more triggers may provide for more sophisticated means of manipulation. In this study, we rationally designed a molecule (*Comp. 1*) capable of responding to two enzymes, alkaline phosphatase (ALP), and reductase. Since the A549 lung cancer cell line showed elevated levels of extracellular ALP and intracellular reductase, we demonstrated that *Comp. 1* responded in a stepwise fashion to those two enzymes and displayed a tandem molecular self-assembly behavior. The selective formation of nanofibers in the mitochondria of the lung cancer cells led to the disruption of the mitochondrial membrane, resulting in an increased level of reactive oxygen species (ROS) and the release of cytochrome C (Cyt C). ROS can react with proteins, resulting in endoplasmic reticulum (ER) stress and the unfolded protein response (UPR). This severe ER stress led to disruption of the ER, formation of vacuoles, and ultimately, apoptosis of the A549 cells. Therefore, *Comp. 1* could selectively inhibit lung cancer cells *in vitro* and A549 xenograft tumors *in vivo*. Our study provides a novel strategy for the selective formation of nanomaterials in lung cancer cells, which is powerful and promising for the diagnosis and treatment of lung cancer.

## 1. Introduction

Nanomaterials [1, 2] are promising for cancer theranostics [3–9], but it has been documented that less than 1% of administered nanomedicines accumulate in tumors [10], thus leading to poor therapeutic efficacy of nanomedicines [11]. It remains a challenge to develop novel strategies to boost the therapeutic efficacy of nanomedicines. Recently, the *in situ* formation of nanomaterials in cancer cells and tumors has emerged as a promising strategy for cancer diagnosis and therapy due to the enhanced selectivity, permeation, and retention of the nanomaterials in tumors [12–20]. The successful examples that have been reported now primarily rely on using a single trigger to control the formation of the nanomaterials *in situ*. For example, nanofibers have been selectively formed in different kinds of cancer cells by the overexpression of enzymes, including alkaline phosphatase (ALP), matrix metalloproteinase (MMP), transglutaminase, and cathepsin B [21–32]. The combination of two or more triggers to form nanomaterials *in situ* may provide for more sophisticated means of control and manipulation, but this strategy has been reported only rarely [13, 33]. Lung cancer cells, including A549 cells, show elevated expression levels of both extracellular ALP and intracellular reductase [34–36]. Taking advantage of these two overexpressed enzymes in A549 cells, we reported, in this study, a peptide derivative capable of responding to these two enzymes and showing a selective tandem molecular self-assembly in A549 cells.

## 2. Results

### 2.1. Molecular Design and Compound Synthesis

**Figure 1:**
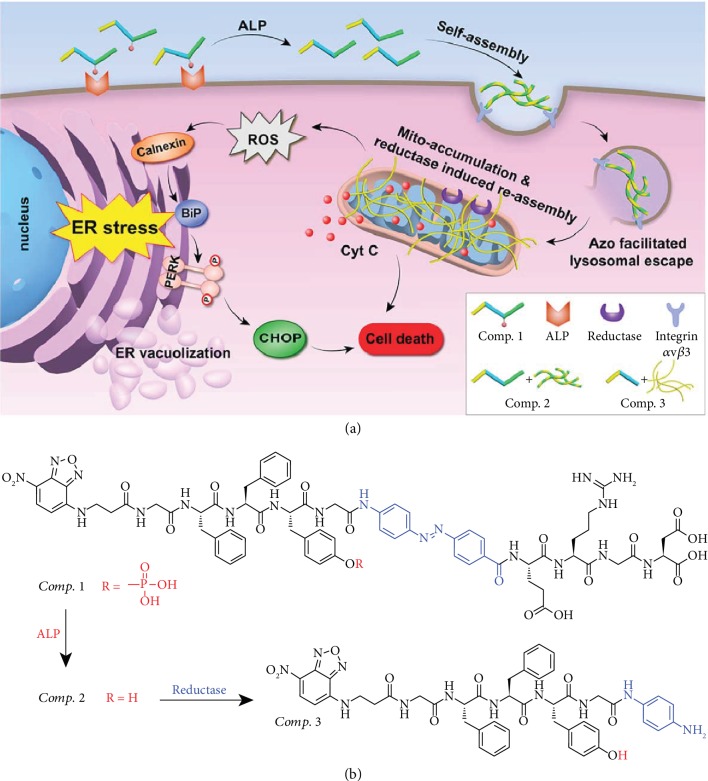
(a) Schematic illustration of the induction of endoplasmic reticulum stress by the tandem molecular self-assembly of *Comp.* 1. (b) Chemical structures and enzymatic conversions from *Comp.* 1 to *Comp.* 2 by ALP and then *Comp.* 2 to *Comp.* 3 by reductase.

In our pilot study, we reported a tandem molecular self-assembly controlled by ALP and glutathione (GSH), specifically in liver cancer cells [13]. We opted to design molecules capable of selectively self-assembling and forming nanomaterials in other types of cancer cells [22, 23, 37]. Most cancer cells exhibit high expression levels of extracellular ALP, which has been widely used for the formation of nanofibers around and inside cancer cells. In addition, we realized that lung cancer cells also show high expression levels of intracellular reductase. We therefore designed the molecule NBD-GFFpYG-N=N-ERGD (*Comp.* 1 in Figure 1(b)) to be capable of responding to both ALP and reductase. We hypothesized that the conversion from *Comp.* 1 to NBD-GFFYG-N=N-ERGD (*Comp.* 2) by extracellular ALP might lead to the formation of nanoparticles or short nanofibers, which could be efficiently taken up by cells through endocytosis. The existence of the azo group in *Comp.* 2 could facilitate lysosomal escape and mitochondrial accumulation of the nanomaterials [38, 39]. Following mitochondrial accumulation, the reductase in the mitochondrial membrane could convert *Comp.* 2 to NBD-GFFYG-aniline (*Comp.* 3 in Figure 1(b)), which could self-assemble into nanofibers in the mitochondria, leading to the disruption of the mitochondrial membrane and the release of cytochrome C (Cyt C), as well as the induction of oxidative stress, which can produce reactive oxygen species (ROS). The ROS could ultimately increase the ER stress and activate the unfolded protein response (UPR), leading to the selective cell death of the lung cancer cells.

The synthesis of *Comp. 1* was simple and straightforward. We first synthesized the Fmoc-protected molecule containing an azobenzene (*Comp. S2* in [Supplementary-material supplementary-material-1]) that could be directly used for standard Fmoc-solid phase peptide synthesis (SPPS). The *Comp. 1* was then obtained through standard SPPS using tritylchloride resin and purified by reversed-phase high-performance liquid chromatography. We also synthesized several control compounds via similar procedures, including NBD-GFFYG-N=N-ERGD (only reductase-responsive *Comp. 2*), NBD-GFFpYGERGD (only ALP-responsive *Comp. 4*), and NBD-GFFYGERGD (non-ALP- and non-reductase-responsive *Comp. 5*).

### 2.2. Dual Enzyme-Triggered Tandem Molecular Self-Assembly

**Figure 2:**
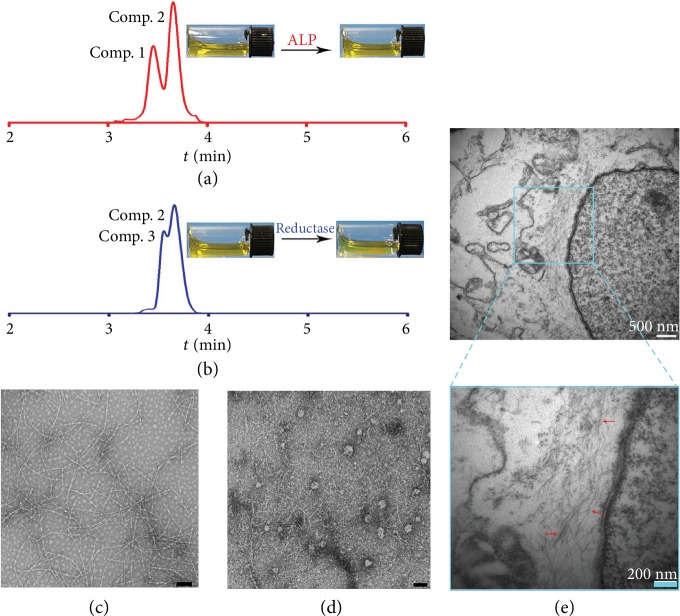
LC spectrum and optical images to indicate (a) the conversion from *Comp.* 1 to *Comp.* 2 by ALP (1 U/mL) at the 2 h time point and (b) the conversion from *Comp.* 2 to *Comp.* 3 by rat liver microsomes (226 *μ*g/mL) and NADPH (50 equiv.) at the 8 h time point. Optical TEM images of (c) the solution formed by adding ALP to *Comp.* 1 (200 *μ*M) for 24 h and (d) the precipitate formed by adding rat liver microsomes and NADPH to *Comp.* 2 for 24 h (scale bars represent 100 nm). (e) Ultrathin sections of A549 cells at 6 h time point post administration of *Comp.* 1; the red arrow points to nanofibers.

The *Comp. 1* could form a clear solution (Figure 2(a)) in phosphate buffer saline (PBS, pH = 7.4) at a concentration of 200 *μ*M (0.03 wt%), which was below its critical aggregation concentration (CAC = 263.6 *μ*M, see the Supporting Information). The transmission electron microscopy (TEM) images revealed amorphous structures in the PBS solution of *Comp. 1*. These results indicated that *Comp. 1* did not self-assemble into regular nanostructures at the concentration of 200 *μ*M. We thereafter added the enzyme ALP (1 U·mL^−1^) to the PBS solution of *Comp. 1* at 37°C, and the solution remained clear after 12 h (Figure 2(a)). The LC-MS trace indicated that over 95% of *Comp. 1* had been converted to *Comp. 2* within 6 h (Figures 2(a) and [Supplementary-material supplementary-material-1]). Accordingly, the nanofibers with a diameter of 6–10 nm formed in the resulting solution of *Comp. 2* (Figure 2(c)). With the addition of rat liver microsomes (226 *μ*g·mL^−1^) and NADPH (50 equiv.) to trigger the conversion from azobenzene to aniline, a yellowish precipitate was clearly observed within 24 h (Figure 2(b)). The LC-MS trace indicated that over 70% of *Comp. 2* had been converted into *Comp. 3* within 24 h (Figures 2(b) and [Supplementary-material supplementary-material-1]). The TEM image exhibited nanofibers with a diameter of 5-7 nm and nanoparticles with a diameter of 30-40 nm in the precipitate. The results obtained by the dodecyl sulfate sodium salt-polyacrylamide gel electrophoresis (SDS-PAGE) indicated that the precipitate consisted of the proteins in the rat liver microsomes and both *Comp. 2* and *Comp. 3* ([Supplementary-material supplementary-material-1]). There were hardly any proteins contained in the supernatant, suggesting that the self-assembling peptide derivatives could form tight complexes with the proteins. The above observations strongly indicated the tandem molecular self-assembly behavior of *Comp. 1* with the catalysis by ALP and reductase.

### 2.3. Intracellular Tandem Molecular Self-Assembly

Generally, cancer cells exhibit higher expression levels of extracellular ALP than normal cells. Before testing the tandem molecular self-assembly of *Comp. 1* in cancer cells, we synthesized the compound TPE-GFFYEG-N=N-EEEE to measure the expression levels of reductase in different cells. Tetraphenylethylene (TPE) is a fluorescent probe with an aggregation-induced emission (AIE) property [37], and TPE-GFFYEG-N=N-EEEE can be converted to TPE-GFFYEG-aniline by reductase. TPE-GFFYEG-aniline could self-assemble into nanostructures and emit a stronger blue fluorescence than TPE-GFFYEG-N=N-EEEE. Therefore, the intensity of blue fluorescence indicated the expression level of reductase in the cells. As shown in Figures [Supplementary-material supplementary-material-1], the A549 cells showed the strongest blue fluorescence in the confocal fluorescence microscopy images of all of the five tested cancer cells (A549, U87, MCF-7, PC-3, and HeLa cells), suggesting that A549 cells exhibited the highest expression level of reductase. The high expression levels of both extracellular ALP and intracellular reductase in A549 cells suggested that the tandem molecular self-assembly of *Comp. 1* might work in A549 cells.

**Figure 3:**
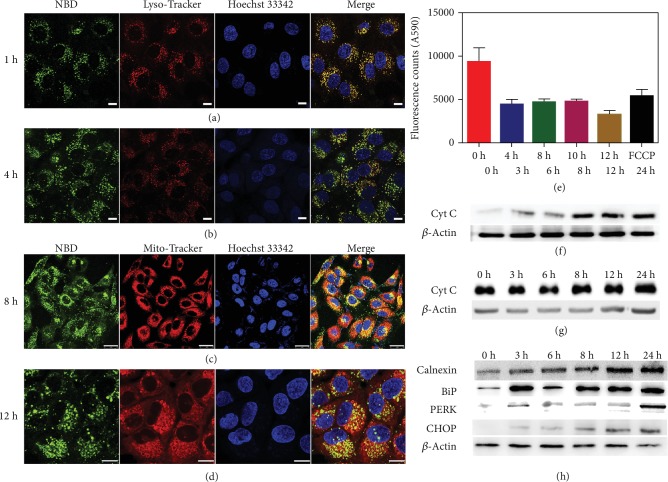
Confocal laser scanning microscopy (CLSM) images of A549 cells treated with *Comp.* 1 (200 *μ*M) for (a) 1 h and then stained with Lyso-Tracker, (b) 4 h and then stained with Lyso-Tracker, (c) 8 h and then stained with Mito-Tracker, and (d) 12 h and then stained with Mito-Tracker (scale bars in (a–c) and (d) represent 25 and 10 *μ*m, respectively). (e) JC-1 assay result in A549 cells treated with *Comp.* 1 and FCCP. Mean ± SEM, *n* = 3. Time-dependent Western blot analysis of cytochrome C (f) from the cytosolic fraction and (g) from the whole-cell fraction of A549 cells treated with *Comp.* 1 (50 *μ*M), and (h) time-dependent Western blot analysis of ER stress-related marker expression in A549 cancer cell after being treated with *Comp.* 1 (50 *μ*M).

To test whether *Comp. 1* could form nanofibers in live cells, Bio-TEM was first used. As shown in Figures 2(e) and [Supplementary-material supplementary-material-1], the ultrathin sections of A549 cells at 6 h post administration of *Comp. 1* (200 *μ*M) displayed nanofibers with the diameters of 4-7 nm in the cytoplasm. These observations clearly indicated the good self-assembly property of our compound in live cells. We then incubated the A549 cells with 200 *μ*M of *Comp. 1* and obtained confocal laser scanning microscopy (CLSM) images of the cells at different time points. As shown in Figures 3(a) and 3(b), there were many green fluorescent dots representing the NBD-peptide within the A549 cells at the 1 h time point. The green fluorescence from NBD colocalized well with the red fluorescence from Lyso-Tracker, which indicated the efficient uptake of the NBD-peptide by cells through endocytosis pathways, in which the self-assemblies could bind to cell membranes by interacting with integrins ([Supplementary-material supplementary-material-1]). However, there was little overlap of the green (NBD) and red (Lyso-Tracker) fluorescence at the 4 h time point, suggesting that the assemblies of NBD-peptide efficiently escaped from the late endosomes/lysosomes. The *Comp. 2* containing the azobenzene group but without the phosphate group had a small fraction of lysosomal escape ([Supplementary-material supplementary-material-1]) at the 4 h time point. Both *Comp. 4* and *Comp. 5* without the phosphate and the azobenzene groups barely escaped from the lysosomes within 4 h. The mean fluorescence intensity in the cells treated with *Comp. 1* and *Comp. 2* was similar, which was significantly higher than that in cells treated with *Comp. 4* and *Comp. 5*. Therefore, it was reasonable to hypothesize that the azobenzene group played a vital role in the cellular uptake and the ability of lysosomal escape of the molecules. We increased the incubation time of cells treated with *Comp. 1* (200 *μ*M) to 8, 10, and 12 h. The results in Figure 3(c) indicated that the green fluorescence from NBD colocalized well with the red fluorescence from the Mito-Tracker in the cytosol at the 8 h time point, suggesting that the NBD-peptide that escaped from the lysosome accumulated in the mitochondria. At 10 and 12 h time points (Figures 3(d), [Supplementary-material supplementary-material-1]), we observed a massive cytoplasmic vacuolization, indicating that the cells were undergoing apoptosis. The occurrence of cytoplasmic vacuolization is generally associated with the dilation of the mitochondria and the ER, which is a classical feature of ER stress. However, as shown in Figures [Supplementary-material supplementary-material-1], *Comp. 2* only caused slight ER stress, and both *Comp. 4* and *Comp. 5* could not induce ER stress.

We performed a time-dependent Western blot assay to monitor the expression levels of Cyt C at different time points. We prepared the cytosolic fraction from A549 cells treated with 50 *μ*M of *Comp. 1* according to an established method for the assay. As shown in Figure 3(f), the concentration of Cyt C in the cytosol, released from mitochondria, increased dramatically during the first 6-8 h and remained at high levels in the following 24 h. JC-1 staining was also used to measure mitochondrial membrane potential. As shown in Figures 3(e) and [Supplementary-material supplementary-material-1], *Comp. 1* caused mitochondrial depolarization at 4 h time point. We also prepared the whole-cell fraction (containing both cytosol and mitochondria) of A549 cells treated with *Comp. 1*; the time-dependent Western blot results indicated that the Cyt C in the whole-cell fraction remained constant during the 24 h (Figures 3(g) and [Supplementary-material supplementary-material-1]). These observations suggested that the self-assembly of *Comp. 1* in the mitochondria led to the disruption of the mitochondrial membrane and the release of Cyt C to the cytosol. Normally, cytoplasmic vacuolization is related to ER stress. We therefore also used a time-dependent Western blot assay to examine the protein expression of ER stress-related signaling markers. As shown in Figures 3(h) and [Supplementary-material supplementary-material-1], calnexin significantly increased after a 12 h treatment with *Comp. 1*, which promoted the unfolded protein response (UPR). The ER chaperone protein BiP, whose expression level was representative of ER stress, was obviously upregulated at the 3 h time point, which correlated well with the lysosomal escape of *Comp. 2* at this time point. BiP was a short-lived protein, and its expression level decreased at the 6 h time point [22, 38]. This protein maintained high expression levels from 8 h to 24 h, representing high levels of ER stress. Since the PERK signaling pathway was activated by BiP, its downstream protein CHOP, a proapoptosis protein, was also upregulated, ultimately leading to cell death [39, 40]. Phosphorylated eIF2*α* (the indicator of PERK-UPR pathway) also was detected by Western blotting, as shown in [Supplementary-material supplementary-material-1], phosphorylation of eIF2*α* began to increase at 6 h, and the expression level increased significantly at 12 h. Taken together, the tandem molecular self-assembly of *Comp. 1* selectively induced death of the non-small-cell cancer cell line (A549 cells) via the release of Cyt C from the mitochondria to the cytosol and the excessive activation of ER stress.

### 2.4. Mechanism Study of ER Stress and Evaluation of Inhibiting Cancer Cells *In Vitro*

**Figure 4:**
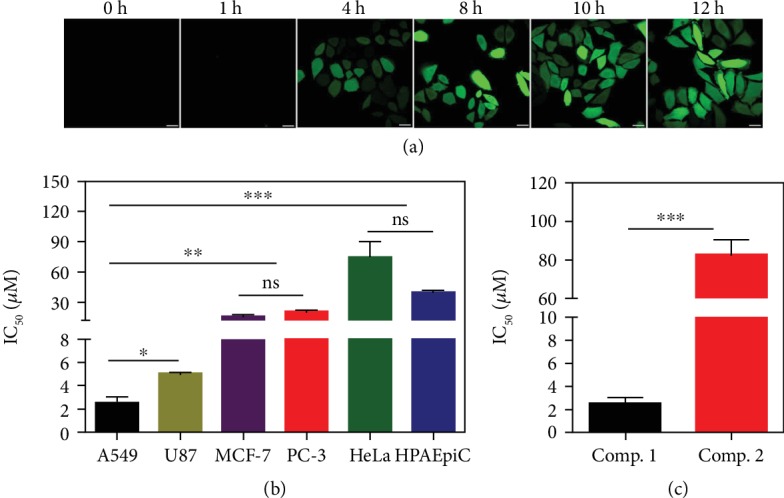
(a) CLSM images of A549 cells to indicate ROS levels measured by H_2_DCFDA at different time points. Scale bars represent 25 *μ*m. (b) IC_50_ values of *Comp.* 1 in different cell lines. (c) IC_50_ of *Comp.* 1 and *Comp.* 2 for A549 cells. Mean ± SEM, *n* = 3. ^∗^*p* < 0.05, ^∗∗^*p* < 0.01, and ^∗∗∗^*p* < 0.001. The data analyzed by Student's *t* test.

As oxidative stress and ER stress are integrally interconnected, ROS can directly disturb the ER protein-folding environment, reduce proper protein folding, and induce UPR activation. Damaged mitochondria can produce oxidative stress, resulting in the production of intracellular ROS. The resulting ROS can react indiscriminately with proteins [40–42]. To understand the mechanism of cytoplasmic vacuolization and cell death, the production of ROS was measured at different time points using H2DCFDA. The A549 cells were incubated with *Comp. 1* (200 *μ*M) at different time points. As the incubation time increased, the amount of ROS produced increased (Figure 4(a)), indicating that *Comp. 1* could cause oxidative stress in the mitochondria and the production of ROS. Pretreating the cells with the antioxidant N-acetyl cysteine (NAC) before treatment with *Comp. 1* could inhibit cytoplasmic vacuolization ([Supplementary-material supplementary-material-1]) and reduce cytotoxicity ([Supplementary-material supplementary-material-1]) and the expression of ER stress markers ([Supplementary-material supplementary-material-1]). Taken together, the tandem molecular self-assembly of *Comp. 1* first disrupted the mitochondrial membrane, subsequently causing oxidative stress and increasing the levels of ROS, ultimately inducing the UPR, endoplasmic reticulum (ER) stress [41, 43, 44], and cell death.

We then investigated the inhibitory capacity of our compounds against different cancer cell lines. As shown in Figures 4(b) and [Supplementary-material supplementary-material-1], *Comp. 1* exhibited an excellent inhibitory capacity in human A549 cells, with a half-maximal inhibitory concentration (IC_50_) of approximately 2.49 *μ*M. The IC_50_ value in A549 cells for the control compound NBD-GFFYG-N=N-ERGD (*Comp. 2*) was 82.7 *μ*M (Figure 4(c)), and the values for NBD-GFFpYGERGD (*Comp. 4*) and NBD-GFFYGERGD (*Comp. 5*) were over 500 *μ*M ([Supplementary-material supplementary-material-1]). The cytotoxicity of *Comp. 1* showed a good selectivity for the A549 cells (IC50 = 2.49 *μ*M), and its IC_50_ values in other cancer cells (U87, MCF-7, PC-3, and HeLa cells) and normal human alveolar epithelial cells (HPAEpiC) were approximately 5.07, 15.86, 20.51, 74.68, and 40.1 *μ*M, respectively. Since there were no significant differences in the cellular uptake of *Comp. 1* by these different cells ([Supplementary-material supplementary-material-1]), the higher toxicity of *Comp. 1* in A549 cells compared with the other cells was probably due to its selective tandem molecular self-assembly in the A549 cells.

### 2.5. Evaluation of Antitumor Efficacy *In Vivo*

**Figure 5:**
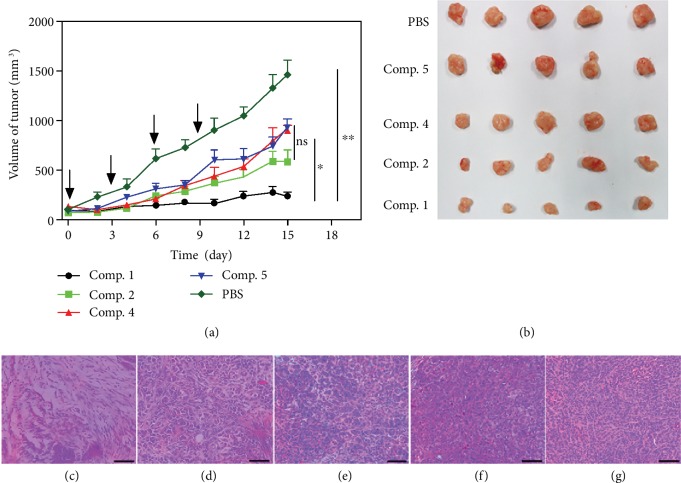
(a) The in vivo anticancer efficacy of different compounds (5.0 mg·kg^−1^, *n* = 5). Mean ± SEM, *n* = 5. ^∗^*p* < 0.05, ^∗∗^*p* < 0.01, and ^∗∗∗^*p* < 0.001. The data analyzed by Student's *t* test. (b) Ex vivo images of tumors extracted from A549 tumor-bearing nude mice at day 15 after being i.v. injected with different compounds. Histological examination of A549 tumors treated with (c) *Comp.* 1, (d) *Comp.* 2, (e) *Comp.* 4, (f) *Comp.* 5, and (g) PBS. Scale bars represent 50 *μ*m.

We further evaluated the in vivo antitumor effects of different compounds in a xenograft tumor model, which was established by the subcutaneous injection of A549 cells in nude mice. When the tumor volume reached approximately 50-100 mm^3^, the mice were treated with *Comp. 1*, *Comp. 2*, *Comp*. *4*, or *Comp. 5* at the same dosage (5 mg kg^−1^, once every three days) via tail vein injection. As shown in Figures 5(a) and 5(b), the final tumor volumes at day 15 in mice treated with *Comp. 1*, *Comp. 2*, *Comp. 4*, and *Comp. 5* were approximately 16.2, 40.0, 61.5, and 62.9%, respectively, compared with that in the PBS group. This result clearly indicated the profound capacity of *Comp. 1* to suppress tumor growth. Meanwhile, the mice treated with *Comp. 1* did not exhibit a loss of body weight, implying the minimal systemic toxicity of *Comp. 1* ([Supplementary-material supplementary-material-1]). We thereafter used hematoxylin-eosin (H&E) staining to evaluate the therapeutic efficacy of the various compounds. As shown in Figures 5(c) and 5(d), the tumor cells in mice treated with *Comp. 1* were severely apoptotic, indicating a great capacity of *Comp. 1* to induce apoptosis in A549 cancer cells in vivo. The tumor cells in mice treated with *Comp. 2* also underwent apoptosis. By contrast, we observed dense nuclei and uniform cytoplasms in the tumor tissues of mice treated with *Comp. 4*, *Comp. 5*, and PBS (Figures 5(e)–5(g)). Overall, these observations demonstrated that *Comp. 1*, possessing a tandem molecular self-assembly ability, could selectively inhibit lung cancer cells and tumors.

## 3. Discussion

In summary, we introduced a compound with selective tandem molecular self-assembly into the A549 lung cancer cell line. The *Comp. 1* responded to extracellular ALP and intracellular reductase in a stepwise manner, showing a tandem molecular self-assembly behavior in the A549 cells. The tandem molecular self-assembly led to the release of Cyt C from the mitochondria to the cytosol, the generation of ROS, and an elevation in ER stress and UPR, successively. Subsequently, the severe ER stress led to disruption of the ER, formation of vacuoles, and ultimately, apoptosis of the A549 cells. We demonstrated that the *Comp. 1* selectively inhibited lung cancer cells both *in vitro* and *in vivo*. Our study provided a novel strategy for the selective formation of nanomaterials in lung cancer cells, which is powerful and promising for the diagnostics and therapy of lung cancer.

## 4. Materials and Methods

### 4.1. Materials

2-Chlorotrityl Chloride Resin (1.1 mmol/g) was purchased from Nankai University resin Co. Ltd. Fmoc-amino acids and o-benzotriazol-1-yl-N,N,N′,N′-tetramethyluronium hexafluorophosphate (HBTU) were obtained from GL Biochem (Shanghai, China). NADPH was bought by Roche Co. Ltd. Rat liver microsomes were purchased from Sigma. Alkali phosphatase (30 U/*μ*L) was obtained from Takara (D2250, Dalian, China) Bio. Inc. PERK antibody (YT3667, Immunoway Co., Ltd.), phospho-eIF2*α* (Ser51) antibody (3398, CST), GRP78 BiP antibody (YT5858, Immunoway), CHOP antibody (15204-1-AP, Proteintech), calnexin antibody (10427-2-AP, Proteintech), and cytochrome C (10993-1-AP). Commercially available reagents and solvents were used without further purification, unless noted otherwise.

### 4.2. General Methods

The synthesized compounds were characterized by 1H NMR (Bruker ARX-400) using DMSO-d6 as the solvent. HPLC was conducted at the LUMTECH HPLC (Germany) system using a C18 RP column with methanol (0.05% of TFA) and water (0.05% of TFA) as the eluents. LC-MS was conducted at the LCMS-20AD (Shimadzu) system. HR-MS was performed at the Agilent 6520 Q-TOF LC/MS using ESI-L low concentration tuning mix (Lot No. LB60116 from the Agilent Tech.). CMC values and size distribution of micelles were determined by dynamic light scattering (DLS); this experiment was conducted on a laser light scattering spectrometer (BI-200SM, Brookhaven, USA). The morphology conversion of the peptide derivatives was measured by TEM performed on a Tecnai G2 F20 system, operating at 200 kV. Cellular uptake and drug tracking images were taken by a confocal laser scanning microscopy (Leica TSC SP8, Germany).

### 4.3. Peptide Synthesis and Characterization

The peptide derivatives were prepared by solid phase peptide synthesis (SPPS) using 2-Chlorotrityl Chloride Resin, and the corresponding N-Fmoc protected amino acids with side chains properly protected by a tertbutyl group (tBu) or 2,2,5,7,8-pentamethyl-chroman-6-sulfonyl (Pmc). 20% piperidine DMF solution was used during deprotection of the Fmoc group. O-Benzotriazol-1-yl-N,N,N′,N′-tetramethyluronium hexafluorophosphate (HBTU) was used as the coupling reagent. Finally, peptide derivative was cleaved using 95% of trifluoroacetic acid with 2.5% of PhSCH_3_ and 2.5% of H_2_O for 30 min. The crude products were purified by high performance liquid chromatography (HPLC) then frozen dried to obtain pure products.

### 4.4. Tandem Molecular Self-Assembly of *Comp. 1*

1.6 mg of *Comp. 1* was dissolved in 500 *μ*L of PBS (pH = 7.4) as a stock solution (PBS was bubbled by Argon for 30 min in advance). The concentration of the stock solution was 200 *μ*M. Na_2_CO_3_ (1 M) was added to the above solution to adjust the final pH to 7.4. The solution was incubated at 37°C. The alkali phosphatase (1 U/mL) was then added to the solution for 12 hours at 37°C to trigger the first step of self-assembly. After that, rat liver microsomes (226 *μ*g/mL) and NADPH (50 equiv.) were added for 24 hours at 37°C to trigger the second step of self-assembly.

### 4.5. Critical Aggregation Concentration (CAC) Determination

The CAC value of compounds *1*, *2*, *4*, and *5* in PBS solution (pH = 7.4) was determined by dynamic light scattering (DLS), and the light scattering intensity was recorded for each concentration analyzed.

### 4.6. SDS-PAGE Experiment

15% polyacrylamide glycine gel was used to analyze protein content. 50 *μ*L of supernatant and precipitate was boiled with SDS, respectively, at 100°C for 10 min. 50 *μ*L of microsomes (226 *μ*g/mL) was used as a control. 10 *μ*L of sample was loaded.

### 4.7. Transmission Electron Microscopy

10 *μ*L of samples were added to the carbon-coated copper grids; excess samples were removed with filter paper, then uranyl acetate for negative staining. At last, samples were placed in the desiccator overnight and observed with transmission electron microscopy.

A549 cells reach to about 70% confluence in 10 cm culture dish, remove the culture medium, and add the fresh culture medium containing 200 *μ*M compound 1. After 4 or 6 h, remove the culture medium, wash the cells by PBS for three times, and scrape the cells with a cell scraper, then centrifuge it for 5 min. The cells were fixed with a 2.5% glutaraldehyde solution at 4°C overnight.

### 4.8. Cell Experiments

PC-3, MCF-7, HeLa, U87, HPAEpiC, and A549 cells were purchased from the Type Culture Collection of the Chinese Academy of Sciences (Shanghai, China); PC-3, MCF-7, HeLa, U87, and A549 cells were cultured in Dulbecco's Modified Eagle's medium (DMEM) supplemented with 10% *v*/*v* fetal bovine serum (FBS), 100 U/mL penicillin, and 100 *μ*g/mL streptomycin; HPAEpiC cells were cultured in RPMI 1640 Medium supplemented with 10% *v*/*v* fetal bovine serum, 100 U/mL penicillin, and 100 *μ*g/mL streptomycin. All cells were at 37°C in a humidified atmosphere of 5% CO_2_.

### 4.9. Reductase Concentration Analysis

We synthesized reductase-based AIE probe TPE-GFFYEG-N=N-EEEE (TPE) to detect its concentration inside different cell lines. Cells were seeded in a CLSM cell culture plate at a concentration of 1 × 10^5^ cell. After incubation for 24 h, upper medium was removed then cell was incubated with TPE (5 *μ*M) for 6 h. The medium was removed, and the cells were washed with DMEM for three times. We then used CLSM to observe fluorescence intensity and used ImageJ to analyze relative fluorescence intensity (*λ*_ex_ = 405 nm, all test conditions are consistent).

### 4.10. Lysosome and Mitochondrial Colocalization

A549 cells was seeded in CLSM cultural dish at a density of 1 × 10^5^ cells. After incubation for 12 h, a medium was removed. The cells were then incubated with *Comp. 1*, *2*, *4*, or *5* (200 *μ*M) for 1 or 4 h (lysosome colocation). Cells were incubated with *Comp. 1* (200 *μ*M) for 8, 10, and 12 h (mitochondrial colocation). Next, the medium containing different compound was removed and washed by PBS for three times, and lysosome tracker (1×) was incubated with cells for 45 min. The medium was removed and the cells were washed by PBS three times. 1-2 mL of DMEM was added for imaging by live cell imaging systems (*λ*_exc._ = 488 nm, emission = 510‐560 nm; *λ*_exc._ = 543 nm, emission = 650‐750 nm). Mito-Tracker (1×) was incubated with cells for 30 min. The medium was then removed and washed by PBS three times. 1-2 mL of DMEM was added for imaging by live cell imaging systems (*λ*_exc._ = 488 nm, emission = 510‐560 nm; *λ*_exc._ = 633 nm, emission = 650‐750 nm, all test conditions are consistent).

### 4.11. JC-1 Staining

Mitochondrial membrane potential was assessed by staining with JC-1 dye (5,50,6,60-tetrachloro-1,10,3,30-tetraethylbenzimidazol-carbocyanine iodide) (abcam). 1 × 10^4^ A549 cells per well, seeded in 96-well plates, were treated with *Comp.1* (200 *μ*M) for different time points and FCCP (100 *μ*M) for 4 h. After treatment, a medium was removed and the cells were then incubated in 100 *μ*L PBS containing JC-1 dye (20 *μ*M) for 15 min at 37°C and subsequently washed with PBS for three times. The red fluorescence (*λ*_exc._ = 543 nm, emission = 570‐620 nm) was determined by a microplate reader (BioTek).

A549 cells were seeded in CLSM cultural dish at a density of 1 × 10^5^ cells. After incubation for 12 h, a medium was removed. The cells were then incubated with *Comp. 1* (200 *μ*M) at different time points. The cells also were incubated with FCCP (100 *μ*M) for 4 h as the positive control group. Then, removing medium, JC-1 staining kit (20 *μ*M) was incubated with A549 cells for 15 min. The medium was removed, and the cells were washed by PBS three times. 1 mL of PBS was added for imaging by live cell imaging systems (green channel (depolarization) *λ*_exc._ = 488 nm, emission = 500 − 540 nm; red channel (hyperpolarization) *λ*_exc._ = 543 nm, emission = 570‐620 nm, all test conditions are consistent).

### 4.12. ROS Detection

The fluorescent dyes H2DCFDA were used to measure the intracellular ROS, cancer cells were incubated with H2DCFDA for 60 min at 37°C in the dark and then washed with PBS thrice. The oxidation of H2DCFDA was detected using a fluorescence microscope.

### 4.13. Time-Dependent Western Bolt

#### 4.13.1. Extracting Cyt C in Cytosol

A549 cells reach to about 50% confluence in 10 cm culture dish, remove the culture medium, and add the fresh culture medium that contains 50 *μ*M compound 1 at different times. At desired time, collect cells by trypsin and centrifuge at 700 g for 5 min at 4 °C. Resuspend cell with 2 mL of PBS then centrifuge at 700 g for 5 min at 4°C. Resuspend cell with 0.2 mL of cytosol extraction buffer containing DTT and protease inhibitors (cocktail and PMSF). Vortex it for 10 s and incubate on ice for 15 min, then centrifuge at 1000 g for 10 min at 4°C, collect the 170 *μ*L supernatant carefully, and discard the pellet then centrifuge at 10,000 g for 30 min at 4 °C. Collect the 150 *μ*L supernatant carefully.

#### 4.13.2. Extracting ER-Related Protein and Cyt C for Whole Cell

A549 cells reach to about 50% confluence in 10 cm culture dish, remove the culture medium, and add the fresh culture medium containing 50 *μ*M compound 1 at different times. At desired time, collect cells by trypsin and centrifuge at 700 g for 5 min at 4°C. Resuspend cell with 2 mL of PBS then centrifuge at 700 g for 5 min at 4°C. Resuspend cell with 0.2 mL of extraction buffer containing DTT and protease inhibitors (cocktail and PMSF). Vortex it for 10 s and incubate on ice for 30 min, then ultrasonic crushing for 1 min. Centrifuge at 10,000 g for 30 min at 4°C. Collect the 150 *μ*L supernatant carefully. Use the BAC kit to quantify the concentration of protein. Perform standard Western blot. Grayscale statistics are processed by Photoshop CS6. Mean ± SEM, *n* = 3.

### 4.14. Cell Proliferation

In this experiment, all cell lines were incubated in a 96-well plate at a density of about 6000. According to previous method [45], cell was fixed by cold 10% trichloroacetic acid solution at 4°C overnight after incubated with compound for 48 h. Then, discarding the fixative solution and washed by water for three times, put the 96-well plate in a 37°C oven to dry. Next, 0.4% SRB solution was used to stain cell for 20 min at room temperature to form protein-bound dry, then using 1% AcOH solution to clean excessive SRB. Put the 96-well plate in a 37°C oven to dry and add 10 mM Tris base solution of 100 *μ*L every well to solubilize the protein-bound dry for 30 min. Measure the OD at 570 nm in a microplate reader.

### 4.15. Cell Uptakes

Cells were incubated in 12-well plates at a density of 10 × 10^6^ cells. The cell cultural medium containing 200 *μ*M of each compound was added to the cells, and the upper medium was removed at 6 h time point. Cells were washed for three times with PBS or DMEM. After being treated with cell lysis solution (1 mL per well contained 200 *μ*L DMSO) for 15 min, the solutions were centrifuged at 14,000 rad/s for 10 min. The amount of compound in the upper solution was determined by a microplate reader at OD of 488 nm (Bio-Rad Mark^TM^, America).

### 4.16. *In Vivo* Antitumor Assay

A549 cells were maintained in our lab. The BALB/c nude (6 weeks old, female) mice purchased from Beijing Vital River Laboratory Animal Technology Co., Ltd. were used. 5 × 10^7^ A549 cells (100 *μ*L) mixed with growth factor-reduced Matrigel (50 *μ*L, BD Biosciences) were subcutaneously injected into the right flank of each mouse. The drug treatment was started when tumor volume reached 80 mm^3^; the tumor volume was calculated by the formula: length∗width^2^/2. Mice (*n* = 5) received compounds 1, 2, 4, and 5 (5.0 mg/kg) in PBS by i.v. injection, whereas the control group (*n* = 5) received PBS only. The compound was injected every three days. The mice were weighed, and tumors were measured every two days during the treatment period.

### 4.17. H&E Staining of Tumor Tissue

The mice were sacrificed to obtain tumor tissues; tumor tissues were fixed by 4% formalin over 48 h, embedded in paraffin, cut into 5-6 *μ*m sections for H&E staining, and evaluated using light microscopy as previously described method.

## Supplementary Materials

Supplementary Materials Figure S1: 1H-NMR spectrum of 3-nitrosobenzoic acid. Figure S2: 1H-NMR spectrum of *Comp.* S1. Scheme S1: synthetic route to *Comp.* S2. Figure S3: 1H-NMR spectrum of Fmoc-Gly-Azo-benzene. Scheme S2: reaction route from *Comp.* S2 to *Comp.* S3 by sodium dithionite (5 equiv.) for 10 min. Figure S4: LC-MS traces to indicate the conversion from *Comp.* S2 to *Comp.* S3 by sodium dithionite (5 equiv.) for 10 min. Figure S5: HR-MS spectrum of *Comp.* 1. Figure S6: HR-MS spectrum of *Comp.* 2. Figure S7: HR-MS spectrum of *Comp.* 4. Figure S8: HR-MS spectrum of *Comp.* 5. Figure S9: HR-MS spectrum of TPE-GFFYEGN=N-EEEE. Figure S10: MALDI-TOF spectrum of TPE-GFFYEGN=N-EEEE. Figure S11: LC traces to show the conversion from *Comp.* 1 to *Comp.* 2 by adding ALP (A) for 1 h and (B) for 6 h; (C) mass spectrum of *Comp.* 2 detected in above process. Figure S12: LC traces to show the conversion from *Comp.* 2 to *Comp.* 3 by adding NADPH (50 equiv.) and rat liver microsomes (226 *μ*g/mL) for (A) 6 h and (B) 24 h; (C) mass spectrum of *Comp.* 3 detected in above process. Figure S13: critical aggregation concentration of (A) *Comp.* 1, (B) *Comp.* 2, (C) *Comp.* 4, and (D) *Comp.* 5. Figure S14: protein content analysis in precipitation and supernatant via SDS-PAGE. Figure S15: TEM images of rat liver microsomes; (A) scale bars represent 500 nm; (B) scale bars represent 250 nm. Figure S16: TEM images of ultrathin sections of A549 cells (A) at 4 h post administration of *Comp.* 1 (200 *μ*M); the red arrow represents nanofiber; (B) without *Comp.* 1, scale bar represents 500 nm; (C) without *Comp.*1, scale bar represents 200 nm. Figure S17: CLSM images of reductase expression detected by TPE (5 *μ*M) for different cell lines; scale bars represent 25 *μ*m. Figure S18: relative concentration of reductase for different cell lines detected by AIE-probe. Mean and standard deviation is plotted for 3 replicates. Figure S19: CLSM images of cells treated with *Comp.* 1 for 4 h in the presence of the RGD (1 mM) (A) scale bars represent 10 *μ*m; (B) scale bars represent 25 *μ*m. Figure S20: CLSM images of A549 cells treated with *Comp.* 2, *Comp.* 4, and *Comp.* 5 (200 *μ*M) for 4 h and then stained with Lyso-Tracker. Scale bars represent 25 *μ*m. Figure S21: confocal laser scanning microscopy images of A549 cells treated with *Comp.* 1 (200 *μ*M) for 10 h and then stained with Mito-Tracker. Scale bars represent 10 *μ*m. Figure S22: morphology of A549 cells treated with *Comp.* 1 (200 *μ*M) for 12 h; the red arrows represent the cytoplasmic vacuolization. Scale bars represent 7.5 *μ*m. Figure S23: CLSM images of A549 cells treated with *Comp.* 2, *Comp.* 4, and *Comp.* 5 (200 *μ*M) for 12 h and then stained with Mito-Tracker. Scale bars represent 7.5 *μ*m. Figure S24: CLSM images of A549 cells stained with Mito-Tracker. Scale bars represent 7.5 *μ*m. Figure S25: CLSM images of JC-1 staining of the A549 cell incubated with *Comp.*1 (200 *μ*M) for different time points and FCCP (100 *μ*M) for 4 h, respectively. Scale bar represents 10 *μ*m. Figure S26: CLSM images of cells treated with *Comp.* 1 (200 *μ*M) for 12 h in the presence of the NAC (40 *μ*M) or NAC (500 *μ*M), scale bars represent 10 *μ*m. Figure S27: the viability of A549 cells in the presence of NAC (500 *μ*M) treated with *Comp.* 1 for (A) 6 h, (B) 12 h, and (C) 24 h. Figure S28: (A) time-dependent Western blot analysis of ER stress-related marker expression in A549 cancer cell after being treated with *Comp.* 1 (50 *μ*M). (B) ER stress signaling in the presence or absence of NAC. Figure S29: (a) the ration of mean gray value quantified from the Western blot of ER stress; (b) the ration of mean gray value quantified from the western blot of Cyt C. Mean ± SEM, *n* = 3. Figure S30: inhibition curve of *Comp.* 1 for different cell lines. Mean ± SEM, *n* = 3. Figure S31: (A) percentage of inhibition for A549 cells incubated with *Comp.*1, *Comp.* 2, *Comp.* 4, and *Comp.* 5, respectively. Mean ± SEM, *n* = 3. Figure S32: cellular uptake of compounds in different cell lines. Mean ± SEM, *n* = 3. Figure S33: (A) weight change of mice treated by different compound and PBS. Mean ± SEM, *n* = 3.
