# Supplementary material for: Tandem Molecular Self-Assembly Selectively Inhibits Lung Cancer Cells by Inducing Endoplasmic Reticulum Stress
Source: Research (Wash D C). 2019 Dec 3;2019:4803624. doi: 10.34133/2019/4803624 (PMC6944487; doi:10.34133/2019/4803624)
Supplement: Supplementary Materials — Figure S1: 1H-NMR spectrum of 3-nitrosobenzoic acid. Figure S2: 1H-NMR spectrum of Comp. S1. Scheme S1: synthetic route to Comp. S2. Figure S3: 1H-NMR spectrum of Fmoc-Gly-Azo-benzene. Scheme S2: reaction route from Comp. S2 to Comp. S3 by sodium dithionite (5 equiv.) for 10 min. Figure S4: LC-MS traces to indicate the conversion from Comp. S2 to Comp. S3 by sodium dithionite (5 equiv.) for 10 min. Figure S5: HR-MS spectrum of Comp. 1. Figure S6: HR-MS spectrum of Comp. 2. Figure S7: HR-MS spectrum of Comp. 4. Figure S8: HR-MS spectrum of Comp. 5. Figure S9: HR-MS spectrum of TPE-GFFYEGN=N-EEEE. Figure S10: MALDI-TOF spectrum of TPE-GFFYEGN=N-EEEE. Figure S11: LC traces to show the conversion from Comp. 1 to Comp. 2 by adding ALP (A) for 1 h and (B) for 6 h; (C) mass spectrum of Comp. 2 detected in above process. Figure S12: LC traces to show the conversion from Comp. 2 to Comp. 3 by adding NADPH (50 equiv.) and rat liver microsomes (226 μg/mL) for (A) 6 h and (B) 24 h; (C) mass spectrum of Comp. 3 detected in above process. Figure S13: critical aggregation concentration of (A) Comp. 1, (B) Comp. 2, (C) Comp. 4, and (D) Comp. 5. Figure S14: protein content analysis in precipitation and supernatant via SDS-PAGE. Figure S15: TEM images of rat liver microsomes; (A) scale bars represent 500 nm; (B) scale bars represent 250 nm. Figure S16: TEM images of ultrathin sections of A549 cells (A) at 4 h post administration of Comp. 1 (200 μM); the red arrow represents nanofiber; (B) without Comp. 1, scale bar represents 500 nm; (C) without Comp.1, scale bar represents 200 nm. Figure S17: CLSM images of reductase expression detected by TPE (5 μM) for different cell lines; scale bars represent 25 μm. Figure S18: relative concentration of reductase for different cell lines detected by AIE-probe. Mean and standard deviation is plotted for 3 replicates. Figure S19: CLSM images of cells treated with Comp. 1 for 4 h in the presence of the RGD (1 mM) (A) scale bars represent 10 μm; (B) [file 4803624.f1.docx]

**Experimental Supporting Information**

**Tandem Molecular Self-assembly Selectively Inhibits Lung Cancer Cells by Inducing Endoplasmic Reticulum Stress**

**Authors**

**Debin Zheng^1^, Yumiao Chen^1^, Sifan Ai^1^, Renshu Zhang^2^, Zhengfeng Gao^3^, Chunhui Liang^1^, Li Cao^1^, Yaoxia Chen^1^, Zhangyong Hong^1^, Yang Shi^1^, Ling Wang^3^, Xingyi Li^2*^ and Zhimou Yang^1, 4*^**

**Affiliations**

^1^Key Laboratory of Bioactive Materials, Ministry of Education, College of Life Sciences, Key Laboratory of Medicinal Chemical Biology, Collaborative Innovation Center of Chemical Science and Engineering, and National Institute of Functional Materials, Nankai University, Tianjin 300071, P. R. China

^2^School of Ophthalmology & Optometry and Eye Hospital, Wenzhou Medical University, and Wenzhou Institute of Biomaterials and Engineering, CNITECH, CAS, Wenzhou, 325035, P. R. China

^3^College of Pharmacy, Nankai University, Tianjin 300071, P. R. China

^4^Jiangsu Center for the Collaboration and Innovation of Cancer Biotherapy, Cancer Institute, Xuzhou Medical University, Xuzhou, Jiangsu, P.R. China

*Correspondence should be addressed to Xingyi Li: [lixingyi_1984@mail.eye.ac.cn](mailto:lixingyi_1984@mail.eye.ac.cn) and Zhimou Yang: yangzm@nankai.edu.cn

**Synthesis and characterization**

Synthesis of 3-nitrosobenzoic acid

To a suspension of 3-amino benzoic acid (10 mmol, 1.37 g) in DCM (50 mL), the solution of Oxone (20 mmol, 12.28 g) in water (100 mL) was added. The mixture was stirred at room temperature for 2 h. The precipitate was filtered and washed by water and ethanol three times respectively. The yellowish solid was obtained. ^1^H NMR (400 MHz, DMSO-d_6_) δ 13.62 (s, 1H), 8.27 (d, J = 8.5 Hz, 2H), 8.05 (d, J = 8.5 Hz, 2H).

***Figure S1*.**  ^1^H-NMR spectrum of 3-nitrosobenzoic acid.

**Synthesis of Comp. S1**

To a solution of benzene-1,4-diamine (10 mmol, 1.37g) and Et_3_N (20 mmol, 1.6 mL), Fmoc-Gly-NHS (10 mmol, 3.94 g) was added. The mixture was stirred at room temperature overnight. The precipitate was filtered and washed by DCM and anhydrous ether three times. Purple solid product was obtained with a yield of 65%. ^1^H NMR (400 MHz, DMSO-d_6_) δ 10.12 (m, 3H), 7.90 (d, J = 7.5 Hz, 2H), 7.69 (m, 5H), 7.42 (t, J = 7.4 Hz, 2H), 7.32 (m, 4H), 4.25 (dt, J = 19.2, 9.4 Hz, 3H), 3.82 (d, J = 6.0 Hz, 2H).

***Figure S2.*** ^1^H-NMR spectrum of ***Comp. S1***

**Synthesis of Comp. S2**

***Scheme S1.*** Synthetic route to ***Comp. S2***.

To a green solution of 3-ntrisobenzoic acid (6 mmol, 906 mg) in DMSO/AcOH (1:1, 48 mL), Comp. S1 was added. The mixture was stirred at room temperature overnight. The reaction was monitored by LC-MS. 30 mL of water was added to quench reaction, and the precipitate was filtered and washed by water three times. The solid product was obtained with a yield of 75% and directly used for next step. ^1^H NMR (400 MHz, DMSO-d_6_) δ 8.12 (d, J = 8.5 Hz, 2H), 7.92 (dt, J = 12.9, 5.1 Hz, 7H), 7.84 (d, J = 9.0 Hz, 2H), 7.72 (dd, J = 12.0, 6.7 Hz, 3H), 7.42 (t, J = 7.2 Hz, 2H), 7.34 (t, J = 7.5 Hz, 2H), 4.28 (m, 3H), 3.86 (d, J = 6.1 Hz, 2H).

***Figure S3.*** ^1^H-NMR spectrum of Fmoc-Gly-Azo-benzene.

***Scheme S2.***  Reaction route from ***Comp. S2*** to ***Comp. S3*** by Sodium dithionite (5 equiv.) for 10 min.

*
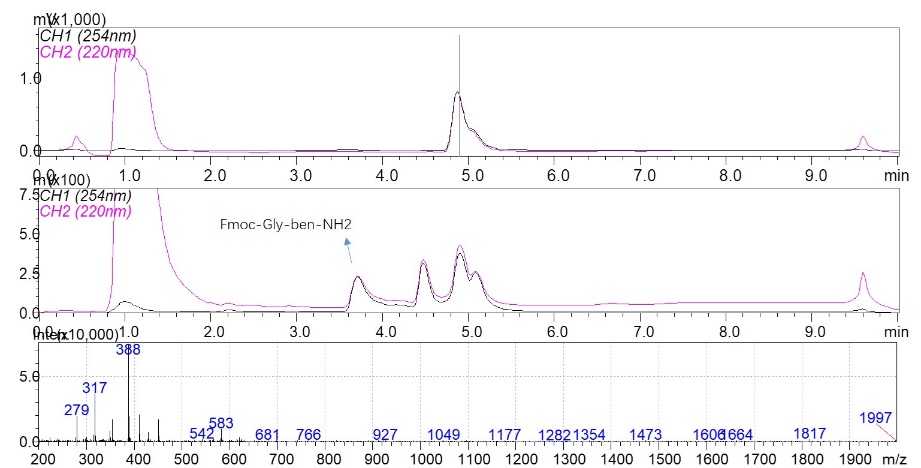
*

***Figure S4.*** LC-MS traces to indicate the conversion from ***Comp. S2*** to ***Comp. S3*** by Sodium dithionite (5 equiv.) for 10 min

**Compound 1:**

HR-MS: calc M^+^=1583.5256, obsvd (M+H)^+^=1584.5297


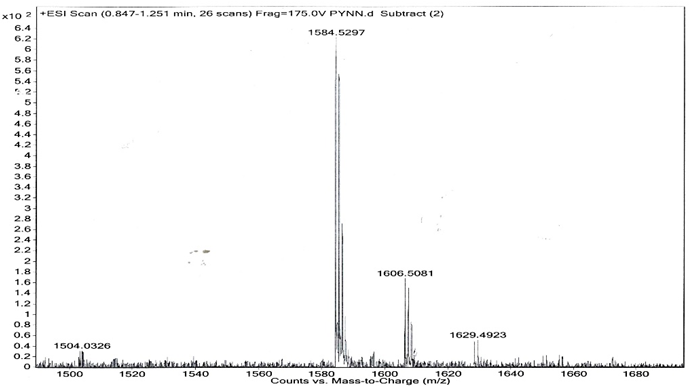


***Figure S5.*** HR-MS spectrum of ***Comp. 1***.

**Compound 2:**

HR-MS: ***Comp. 2***: calc M^+^=1503.5593, obsvd (M+2H) ^+^=1505.5688.


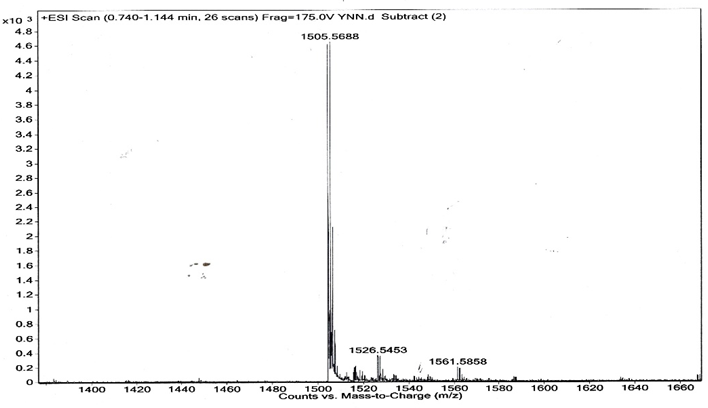


***Figure S6.***  HR-MS spectrum of ***Comp. 2***.

**Compound 4:**

HR-MS: calc M^+^=1360.4510, obsvd (M+H)^+^ =1361.4568.


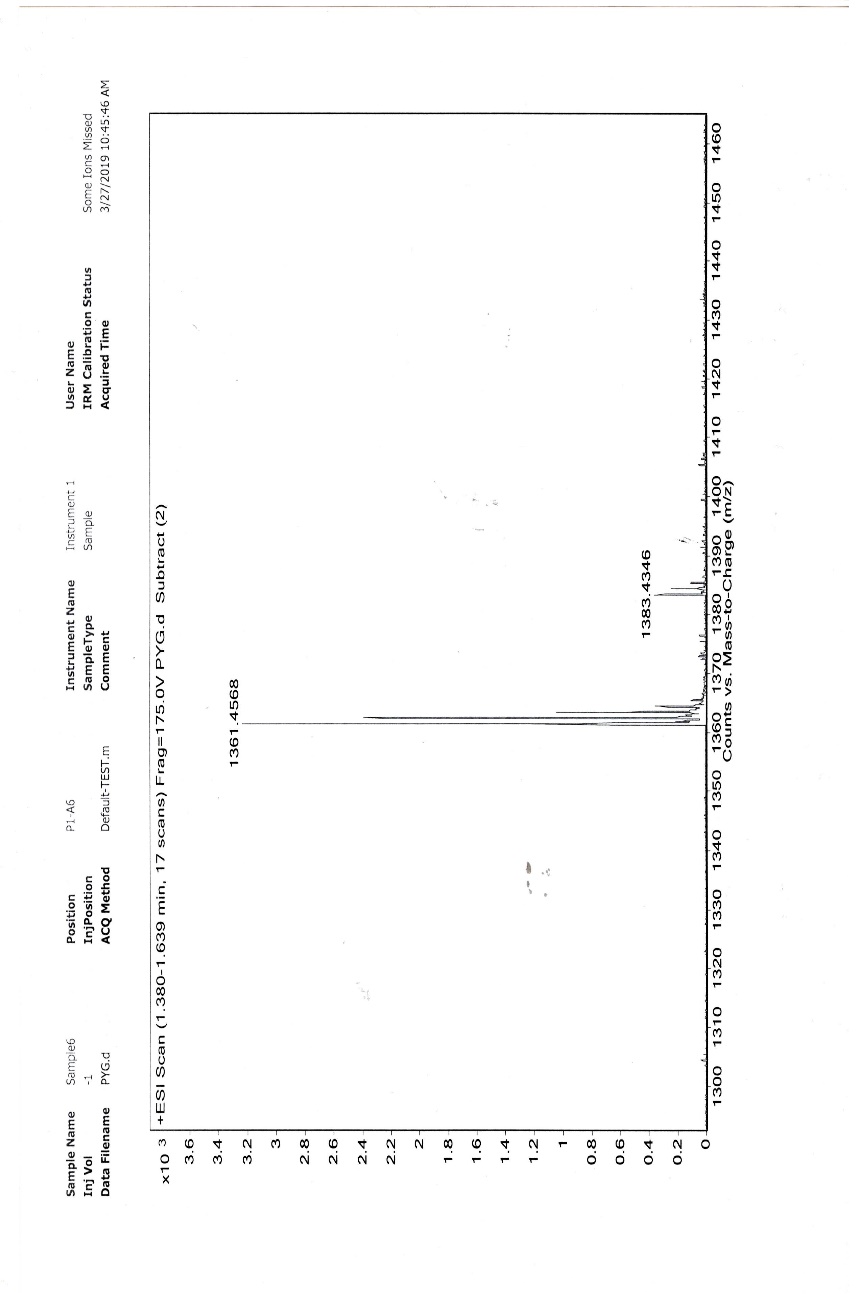


***Figure S7.***  HR-MS spectrum of ***Comp. 4***.

**Compound 5:**

HR-MS: calc M^+^ =1280.4847, obsvd (M+H)^+^ = 1281.4913.


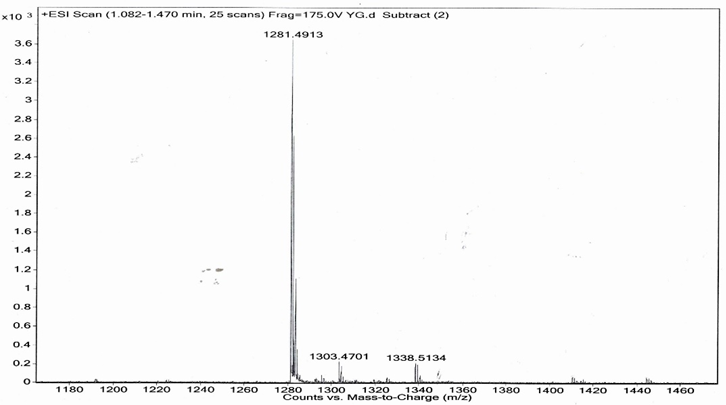


***Figure S8.*** HR-MS spectrum of ***Comp. 5***.

**TPE-GFFYEGN=N-EEEE**

HR-MS: calc M^-^ =1815.6803, obsvd M^2-^/2= 907.3303.


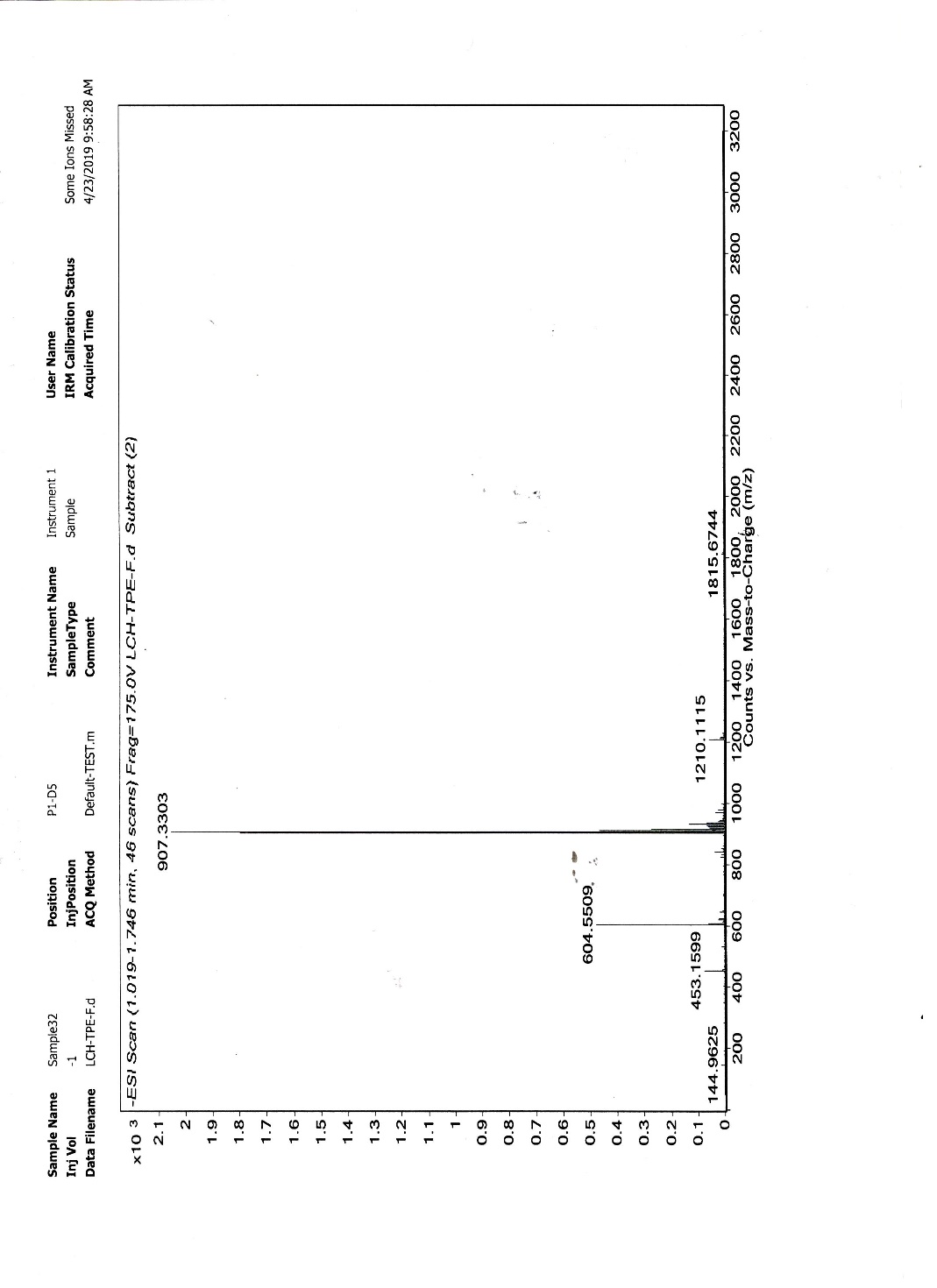


***Figure S9.*** HR-MS spectrum of TPE-GFFYEGN=N-EEEE

MALDI-TOF: calc M^+^ =1816.6803, obsvd (M+Na)^+^ =1838.14

***Figure S10.*** MALDI-TOF spectrum of TPE-GFFYEGN=N-EEEE


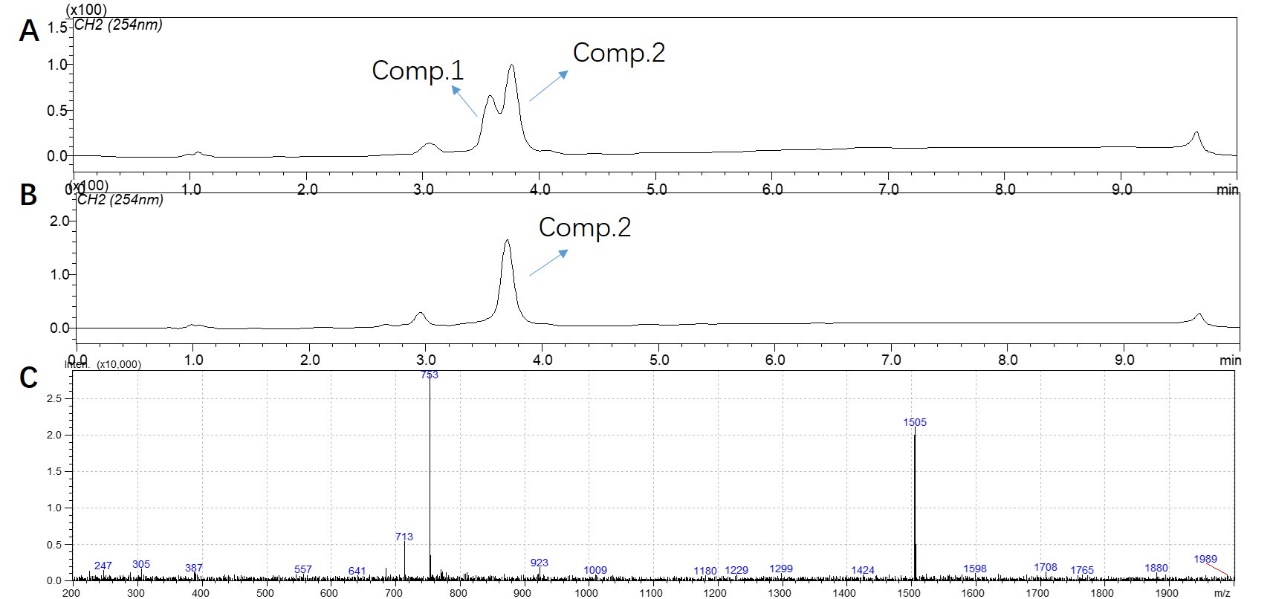


***Figure S11.*** LC traces to show the conversion from ***Comp. 1*** to ***Comp. 2*** by adding ALP (A) for 1 h and (B) for 6 h; (C) mass spectrum of ***Comp. 2*** detected in above process.


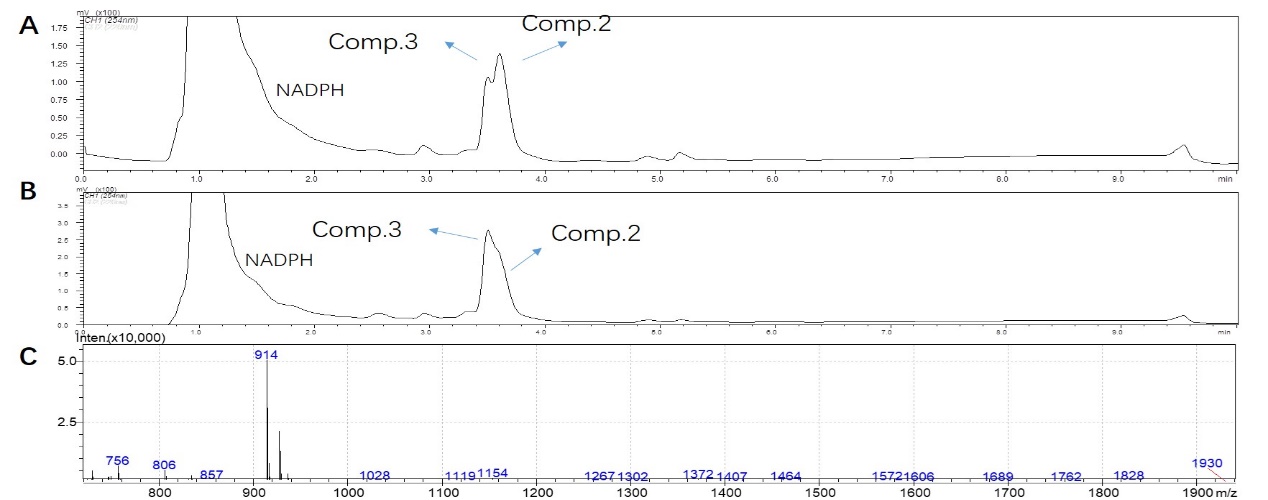


***Figure S12.*** LC traces to show the conversion from ***Comp. 2*** to ***Comp. 3*** by adding NADPH (50 equiv.) and rat liver micsomes (226 μg/mL) for (A) 6 h and (B) 24 h; (C) mass spectrum of ***Comp. 3*** detected in above process.


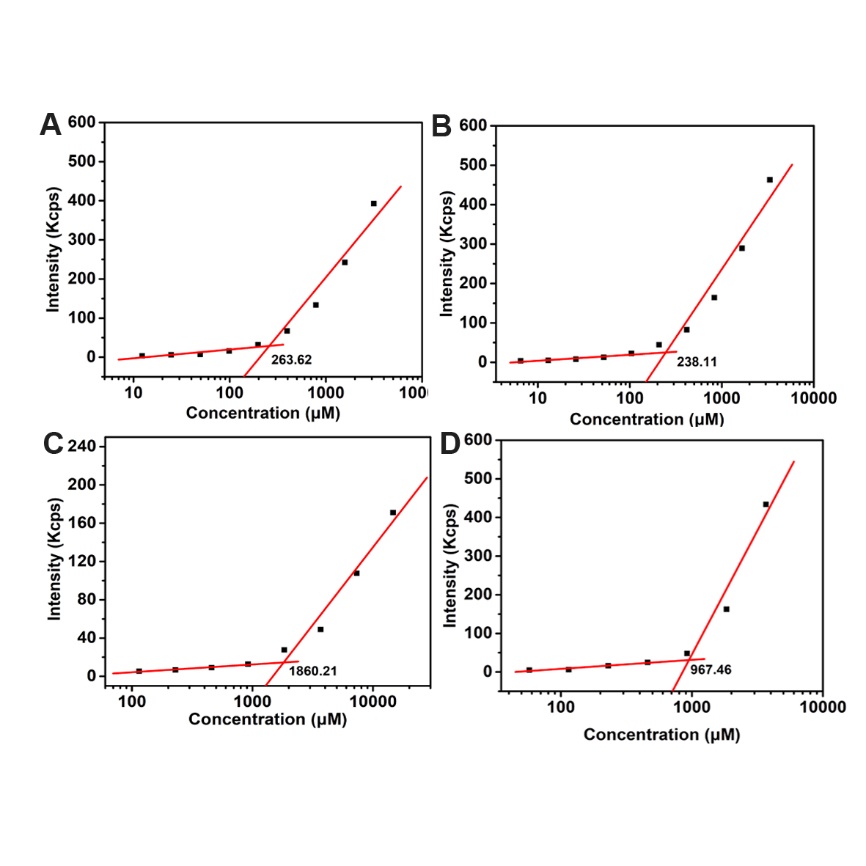


***Figure S13****.* Critical aggregation concentration of (A) ***Comp. 1***, (B) ***Comp. 2***, (C)***Comp. 4***, (D) ***Comp. 5***.


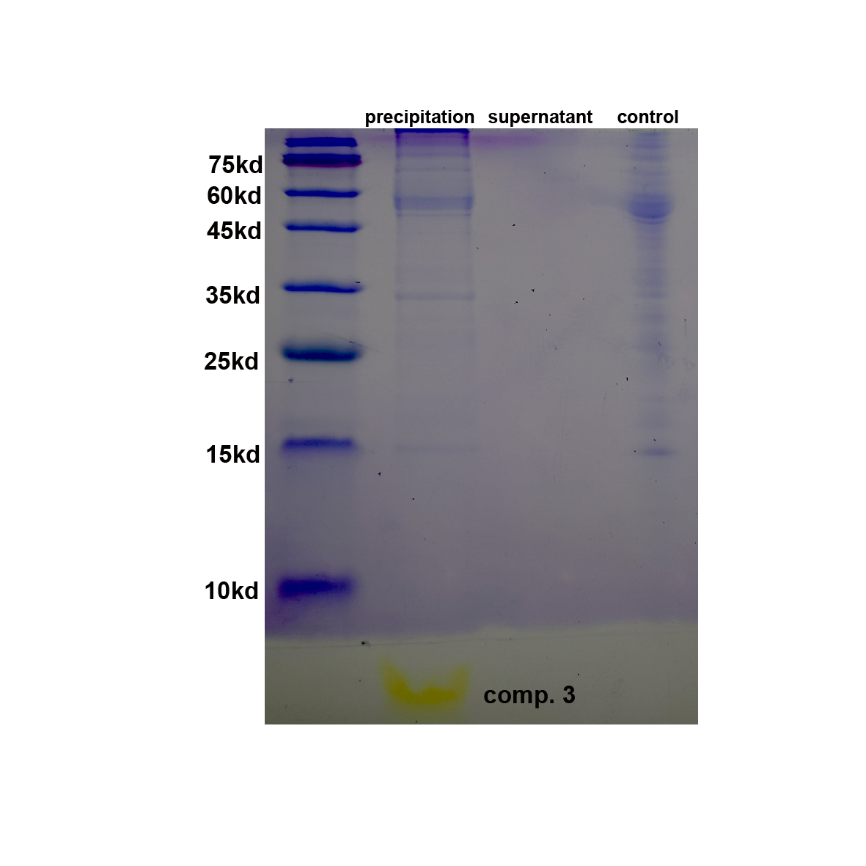


***Figure S14.*** Protein content analysis in precipitation and supernatant *via* SDS-PAGE.


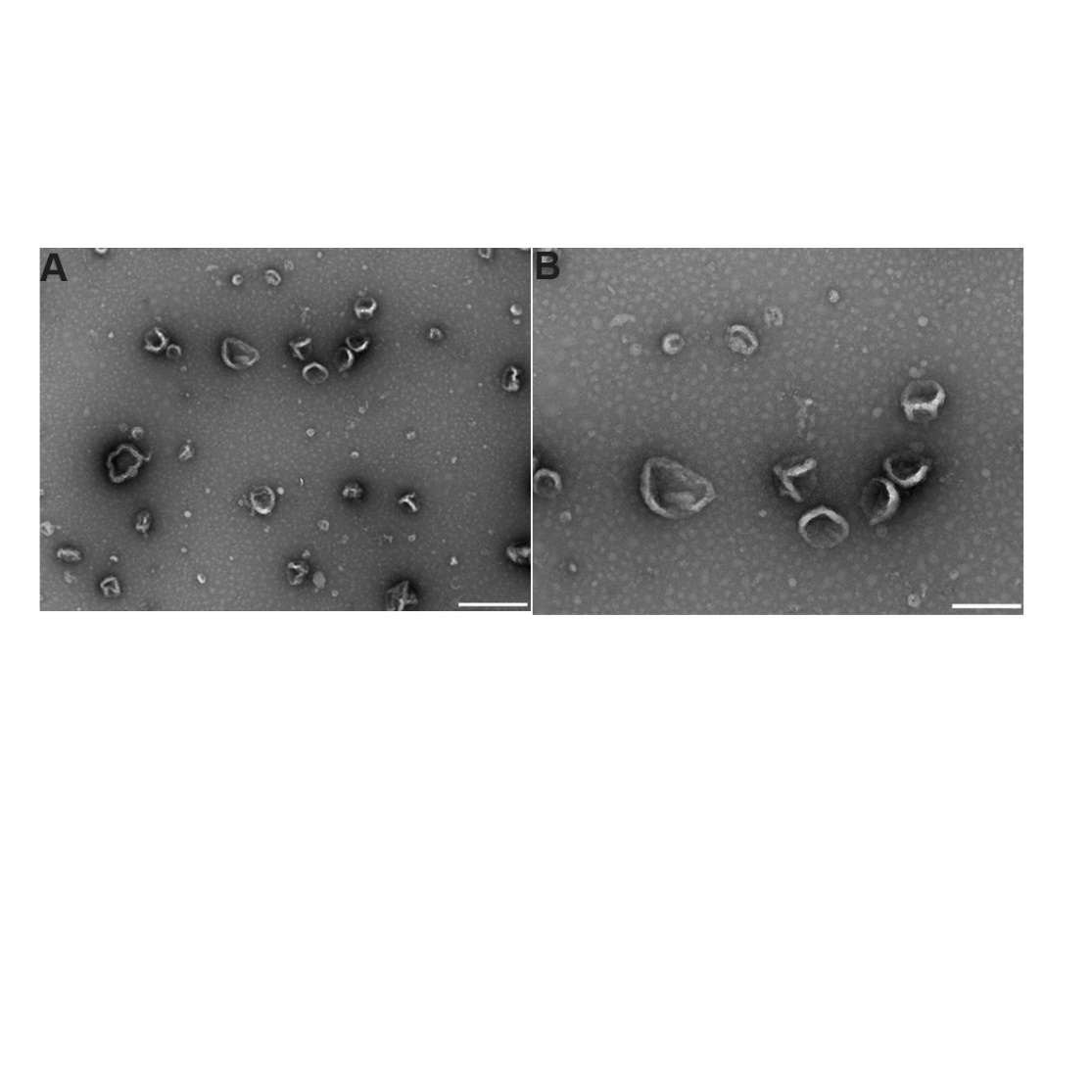


***Figure S15.***  TEM images of rat liver microsomes (A) scale bars represent 500 nm; (B) scale bars represent 250 nm.


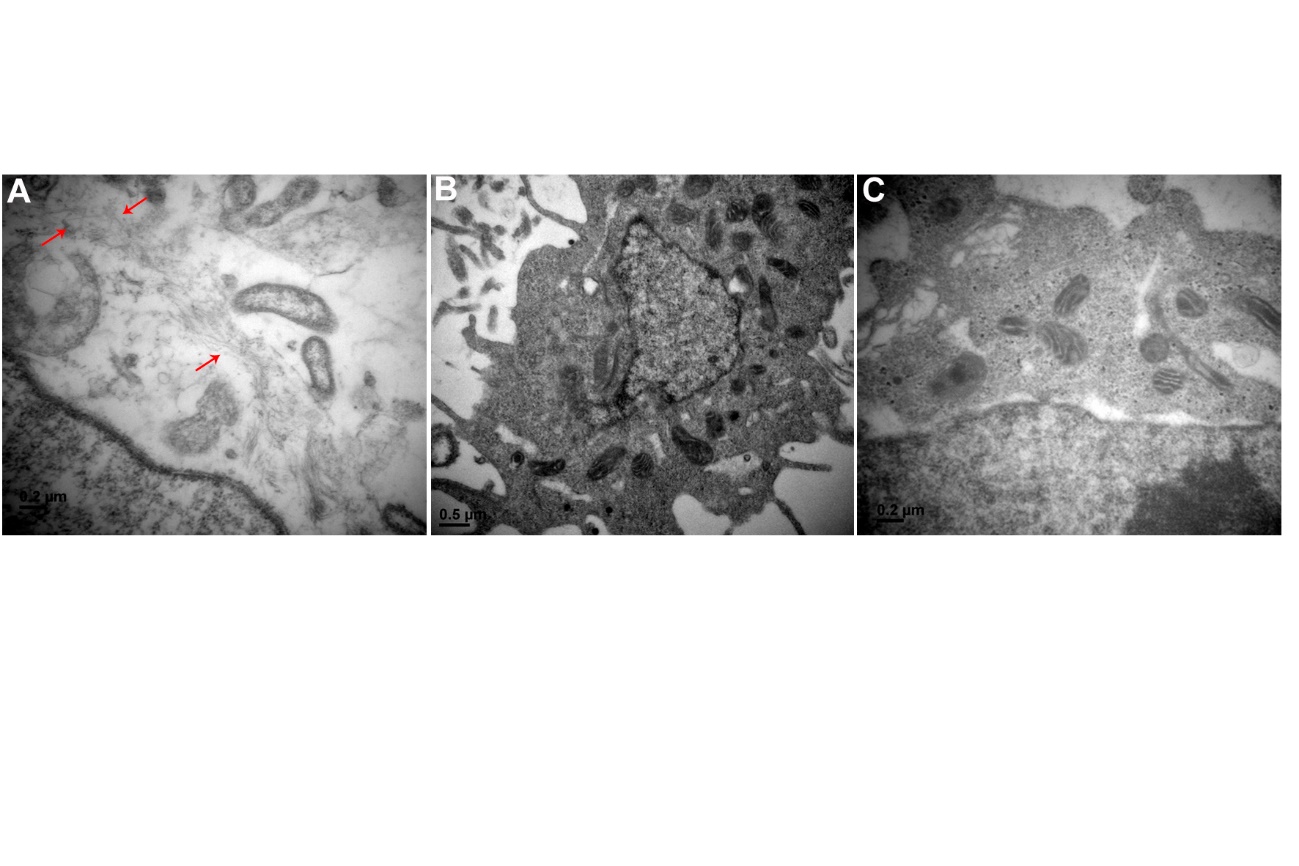


***Figure S16.***  TEM images of ultrathin sections of A549 cells A) at 4 h post administration of ***Comp. 1*** (200μM), the red arrow represents nanofiber; B) without ***Comp.1*** , scale bar represent 500 nm; C) without ***Comp.1*** , scale bar represent 200 nm.


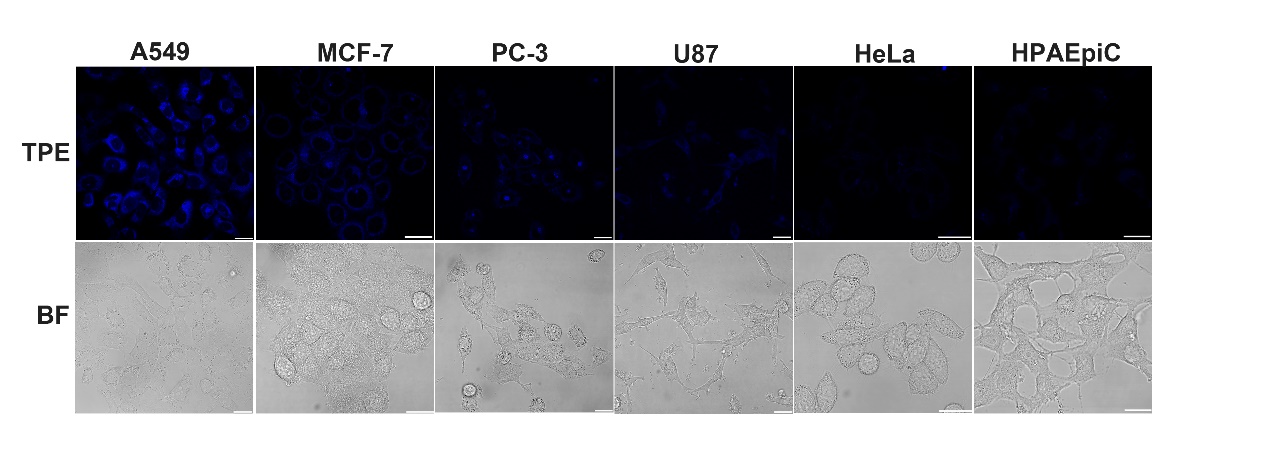


***Figure S17.*** CLSM images of reductase expression detected by TPE(5 μM) for different cell lines, scale bars represent 25 μm.


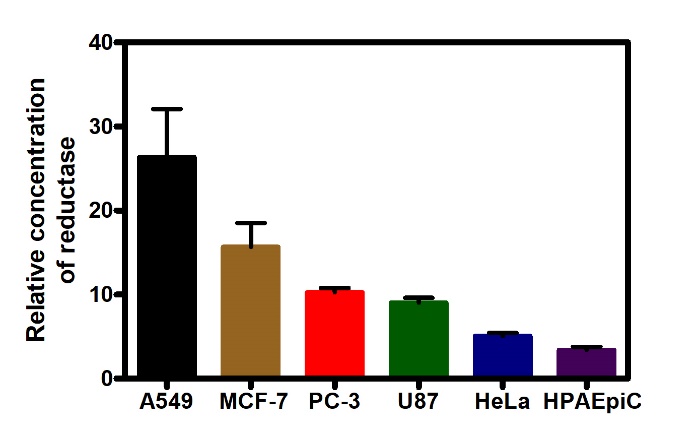


***Figure S18.*** Relative concentration of reductase for different cell lines detected by AIE-probe. Mean and standard deviation is plotted for 3 replicates.


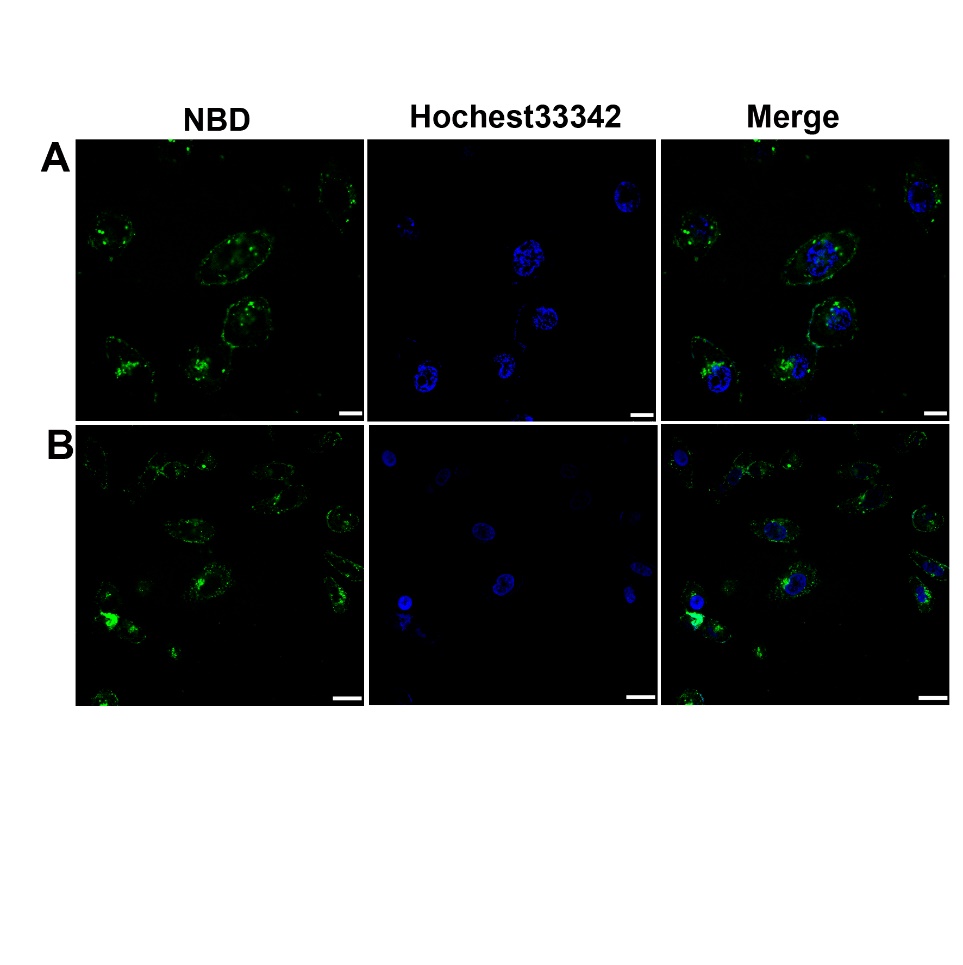


***Figure S19.*** CLSM images of cells treated with ***Comp. 1*** for 4 h in the presence of the RGD (1 mM) (A) Scale bars represent 10 µm; (B) Scale bars represent 25 µm


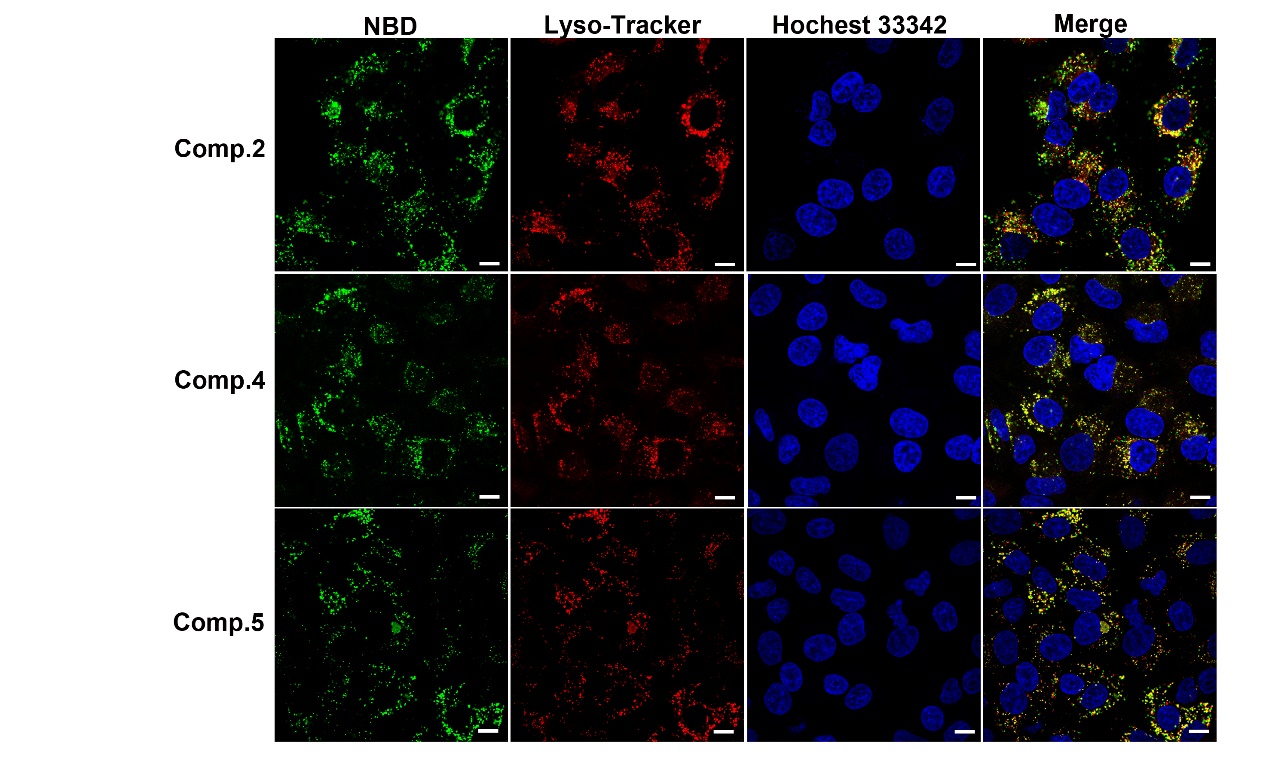


***Figure S20.*** CLSM images of A549 cells treated with ***Comp. 2***, ***Comp. 4*** and ***Comp. 5*** (200 μM) for 4 h and then stained with Lyso-Tracker. Scale bars represent 25 μm.


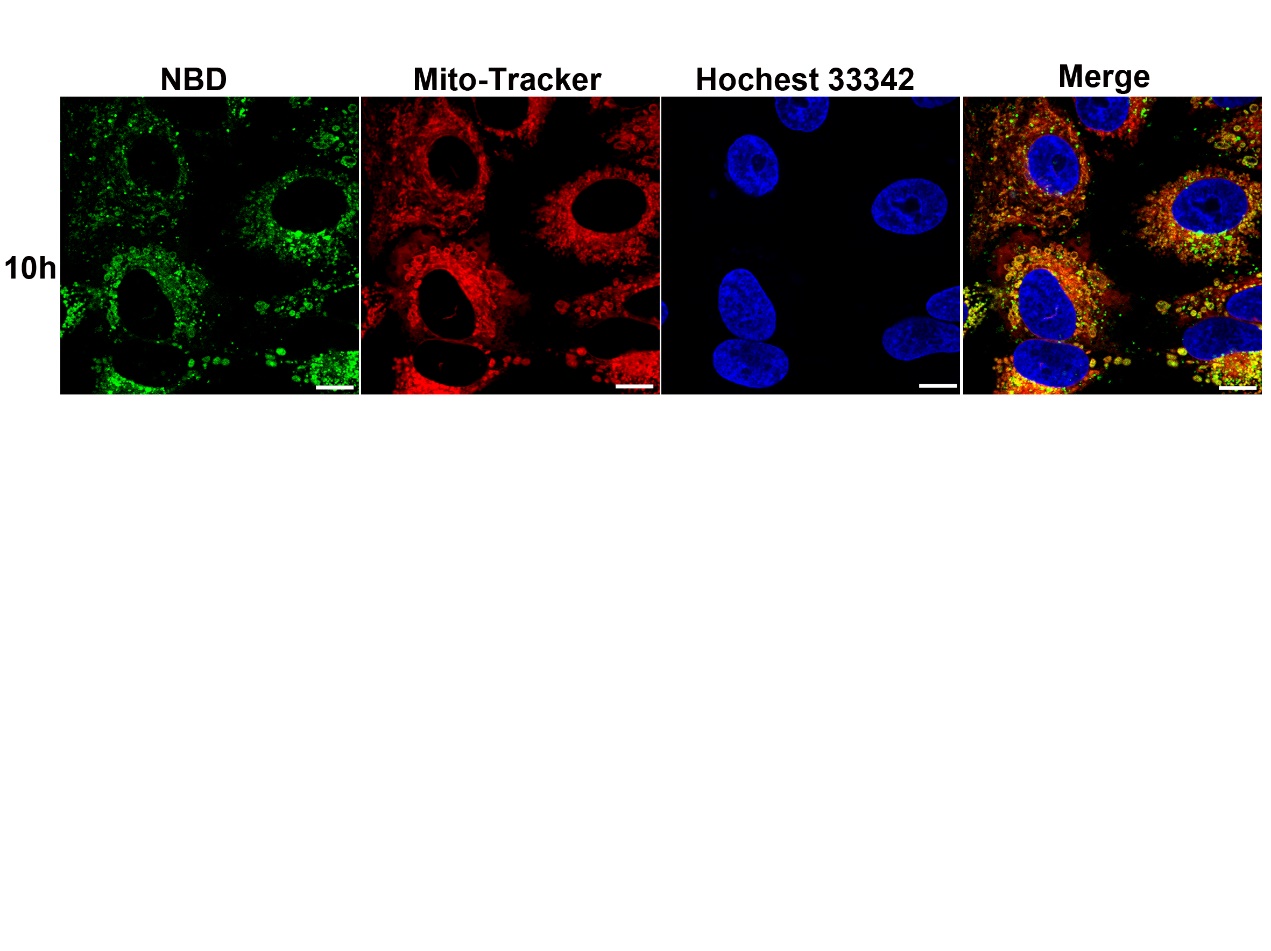


***Figure S21.*** Confocal laser scanning microscopy images of A549 cells treated with ***Comp. 1*** (200 μM) for 10 h, and then stained with Mito-tracker. Scale bars represent 10 μm.


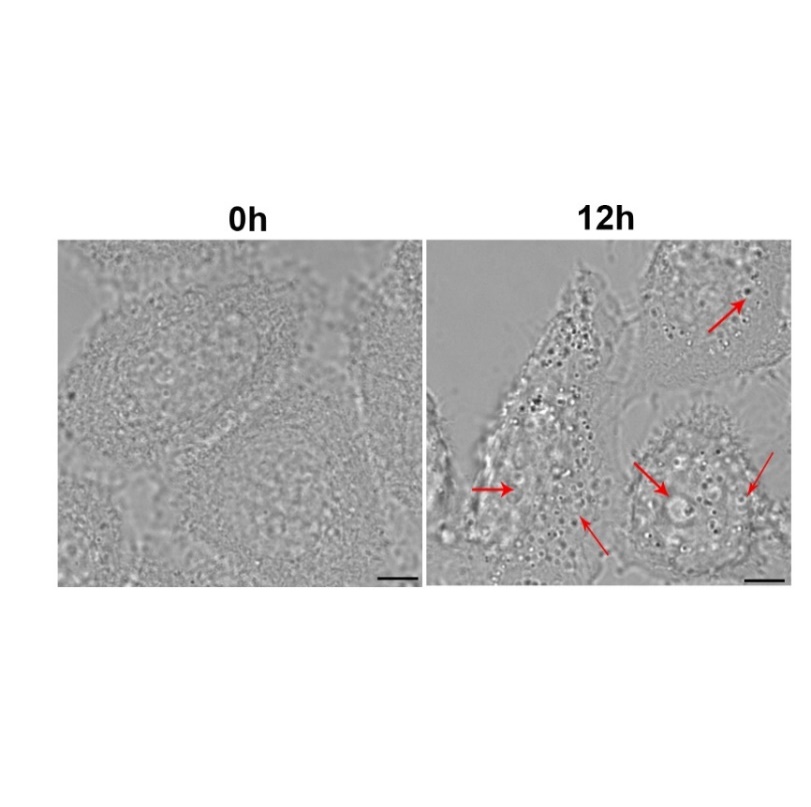


***Figure S22.***  Morphology of A549 cells treated with ***Comp. 1*** (200 μM) for 12 h, the red arrows represent the cytoplasmic vacuolization. Scale bars represent 7.5 μm.


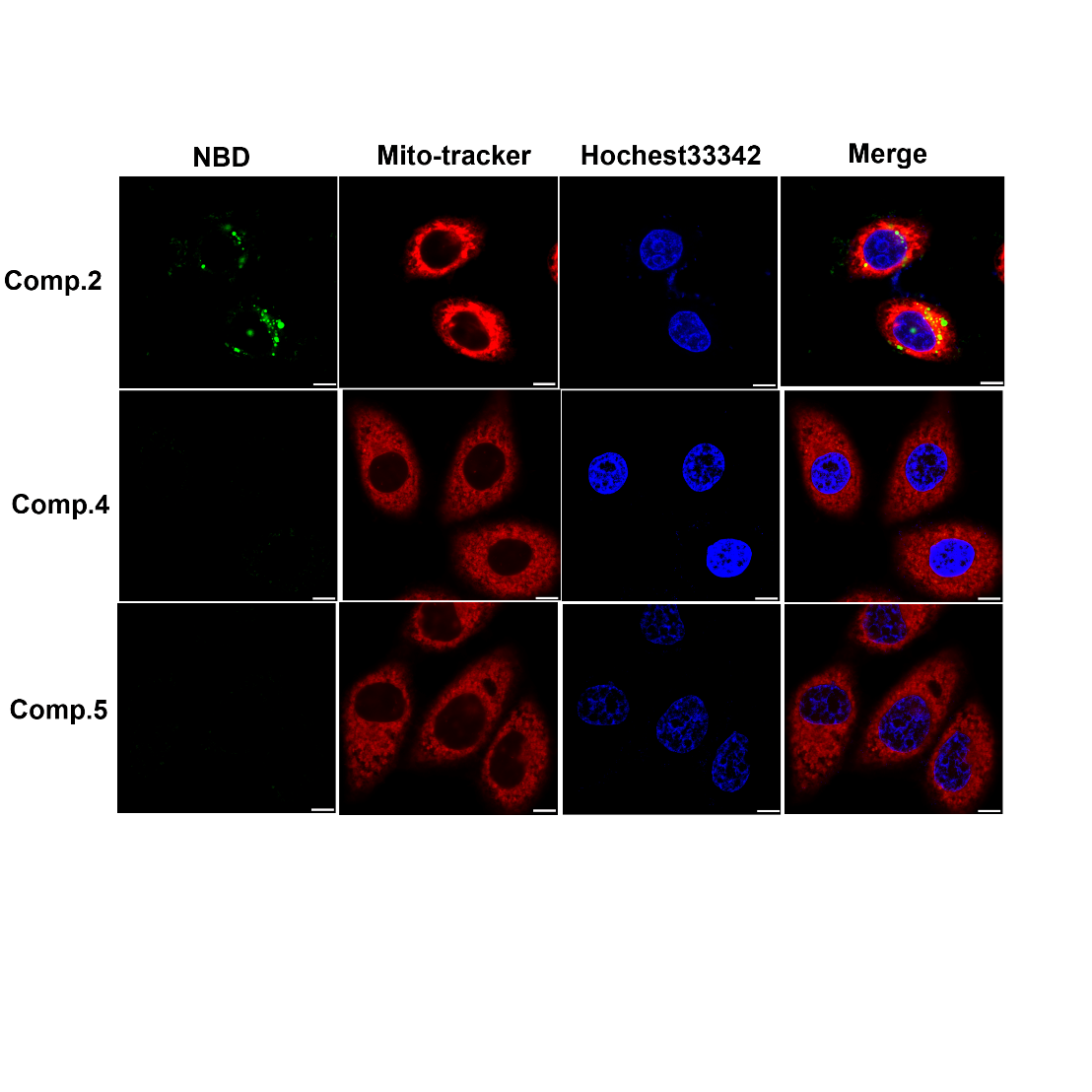


***Figure S23.***  CLSM images of A549 cells treated with ***Comp. 2***, ***Comp. 4*** and ***Comp. 5*** (200 μM) for 12 h and then stained with Mito-Tracker. Scale bars represent 7.5 μm.


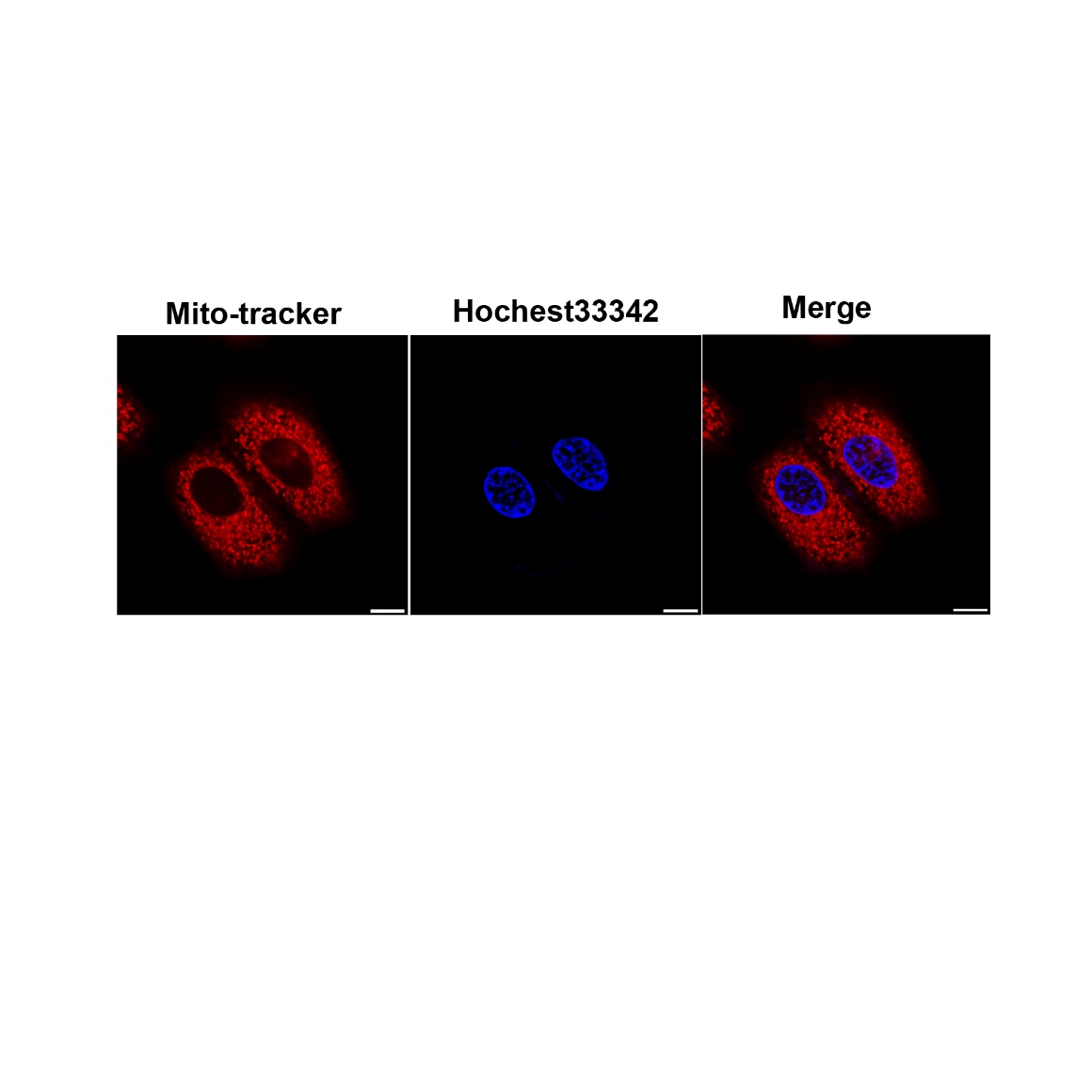


***Figure S24.*** CLSM images of A549 cells stained with Mito-Tracker. Scale bars represent 7.5 μm.


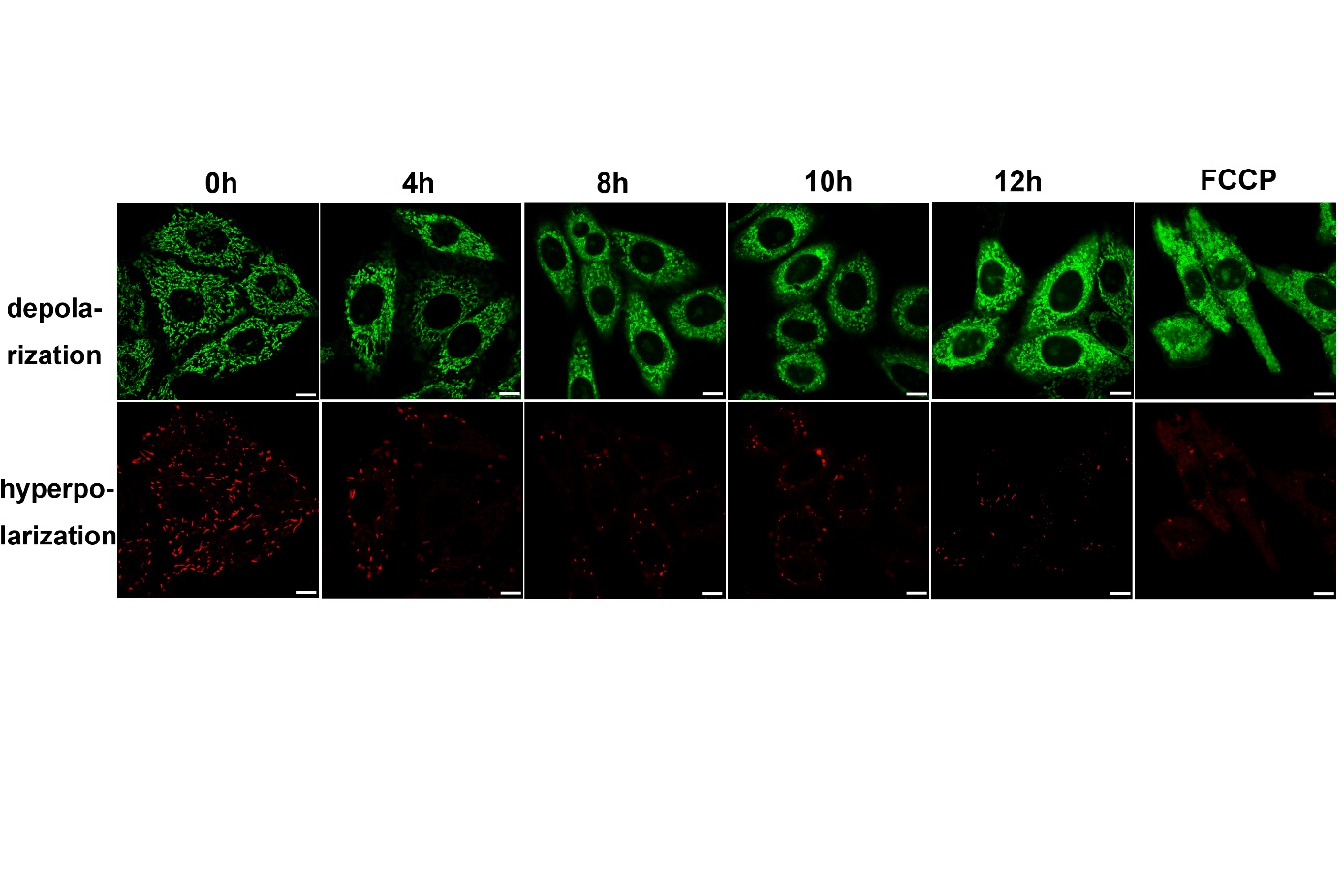


***Figure S25.***  CLSM images of JC-1 Staining of the A549 cell incubated with ***Comp.1*** (200 μM) for different time points and FCCP (100 μM) for 4 h respectively. Scale bar represent 10 μm.


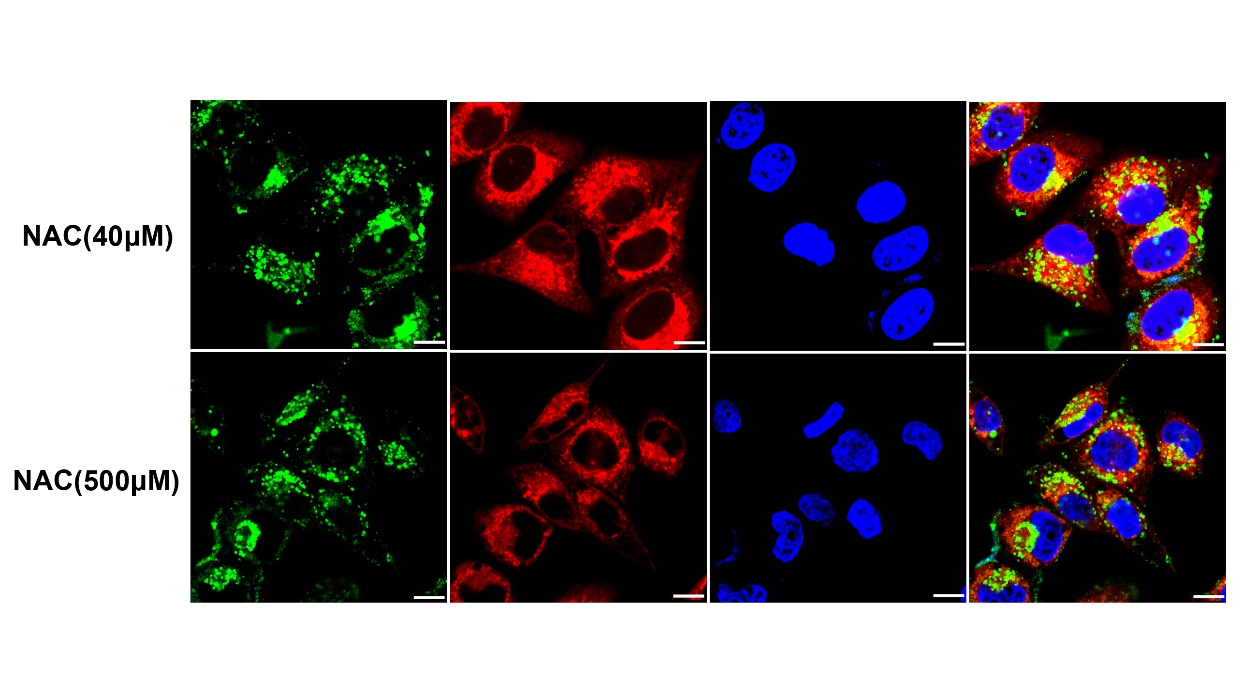


***Figure S26.*** CLSM images of cells treated with ***Comp. 1*** (200 μΜ) for 12 h in the presence of the NAC (40 μM) or NAC (500 μM), scale bars represent 10 µm.


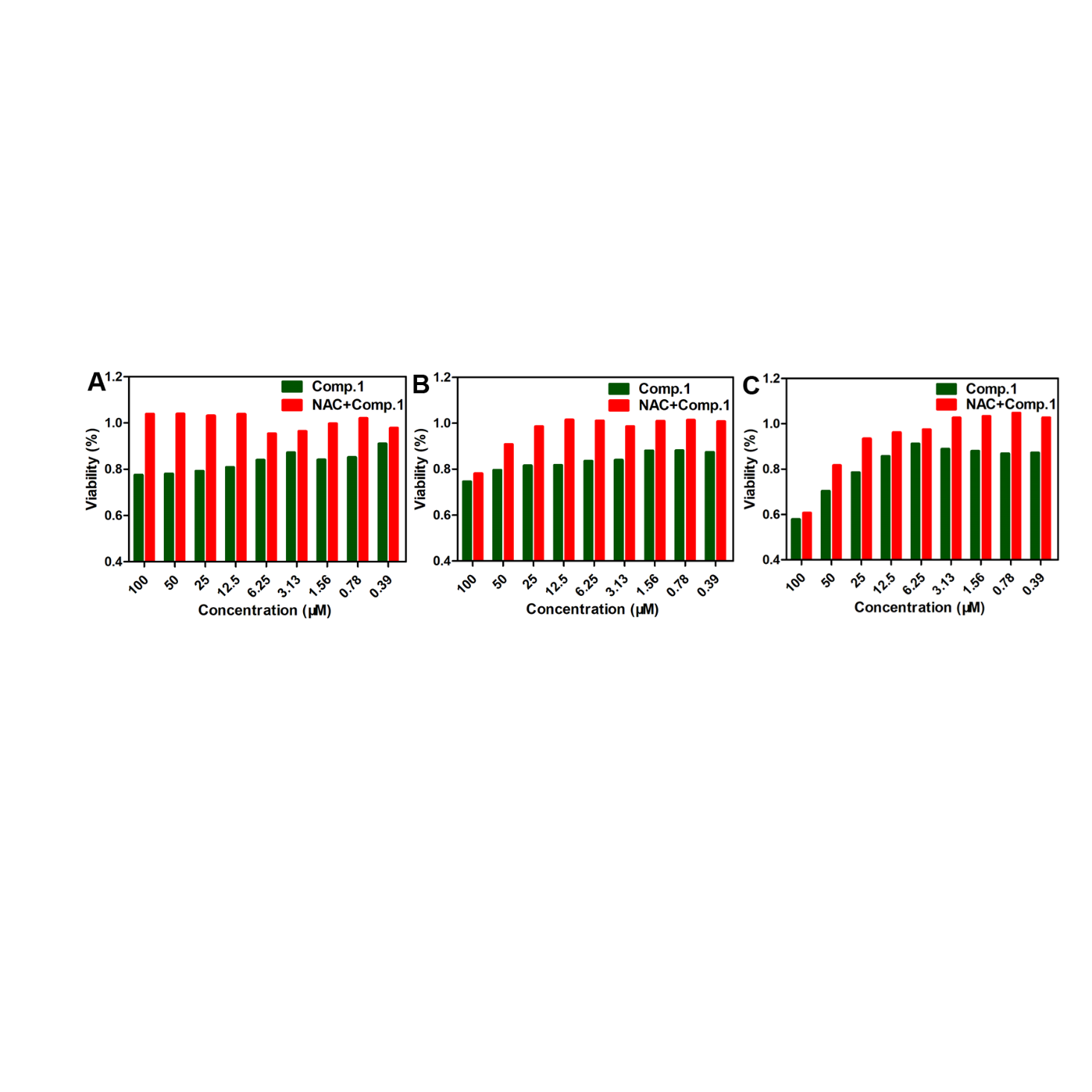


***Figure S27.*** The viability of A549 cells in the presence of NAC (500 μM) treated with ***Comp. 1*** for (A) 6 h; (B) 12 h; and (C) 24 h.


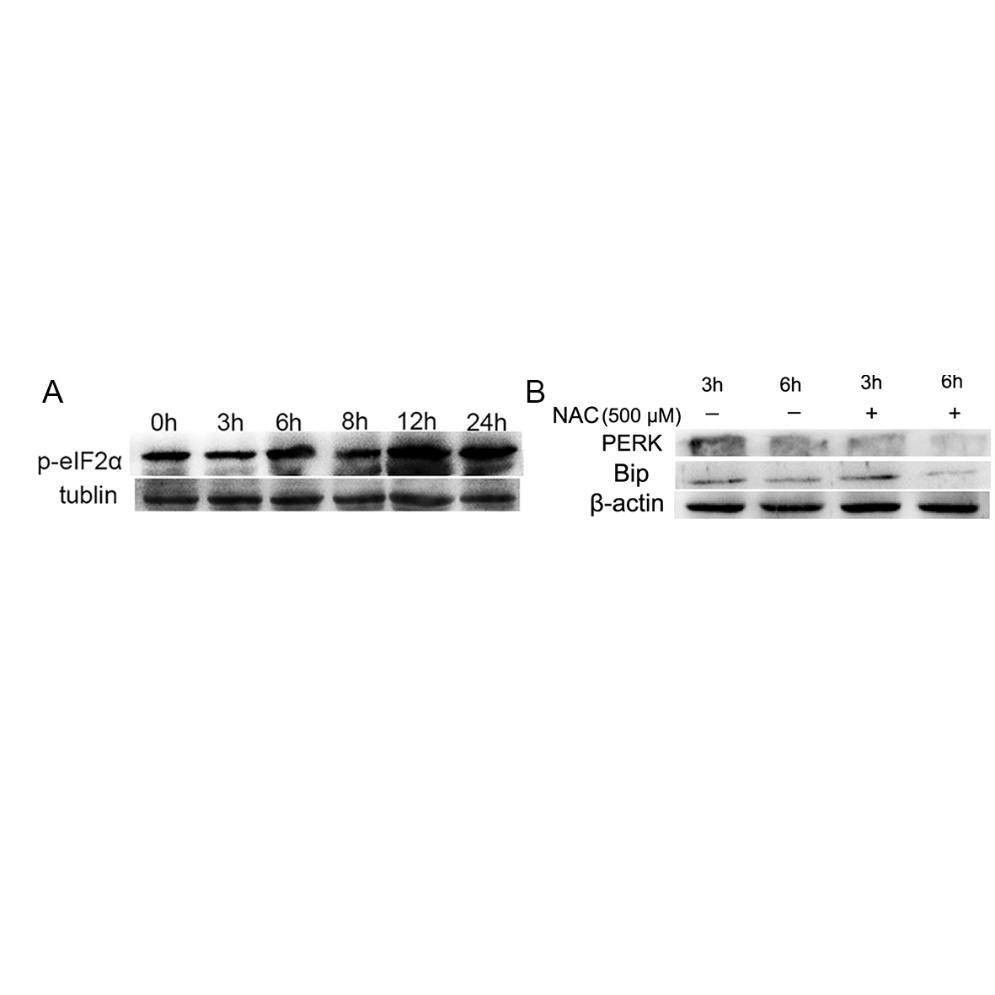


***Figure S28.*** (A) Time-dependent Western blot analysis of ER stress-related marker expression in A549 cancer cell after being treated with ***Comp. 1*** (50 μM). (B) ER stress signaling in present or absent of NAC.

**
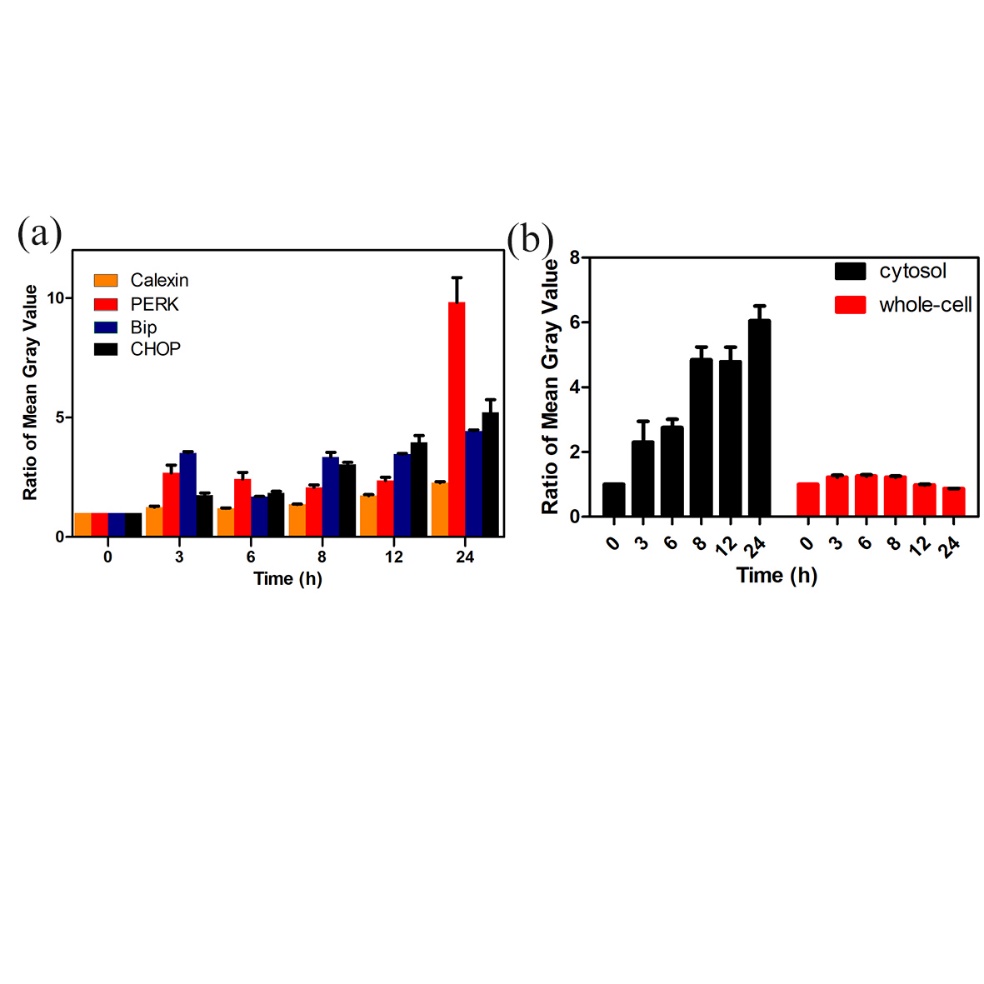
**

***Figure S29.*** （a）the ration of mean gray value quantifid from the western blot of ER stress, (b) the ration of mean gray value quantifid from the western blot of cyto C. *Mean ± SEM, n=3.*


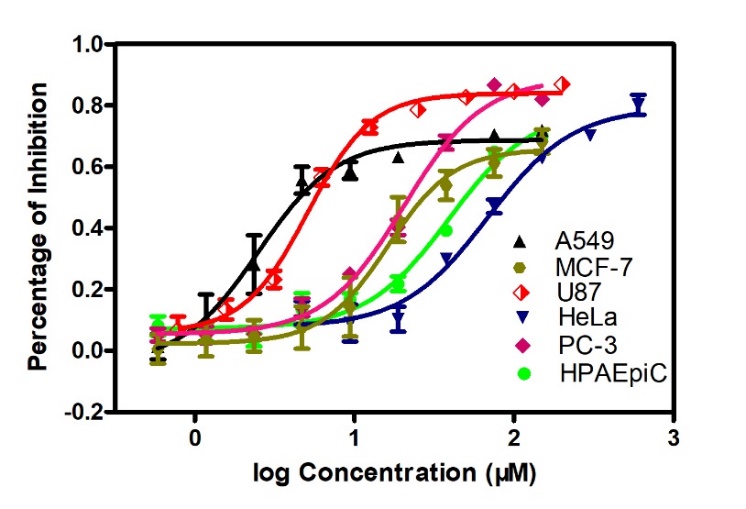


***Figure S30.*** Inhibition curve of ***Comp. 1*** for different cell lines. *Mean ± SEM, n=3.*


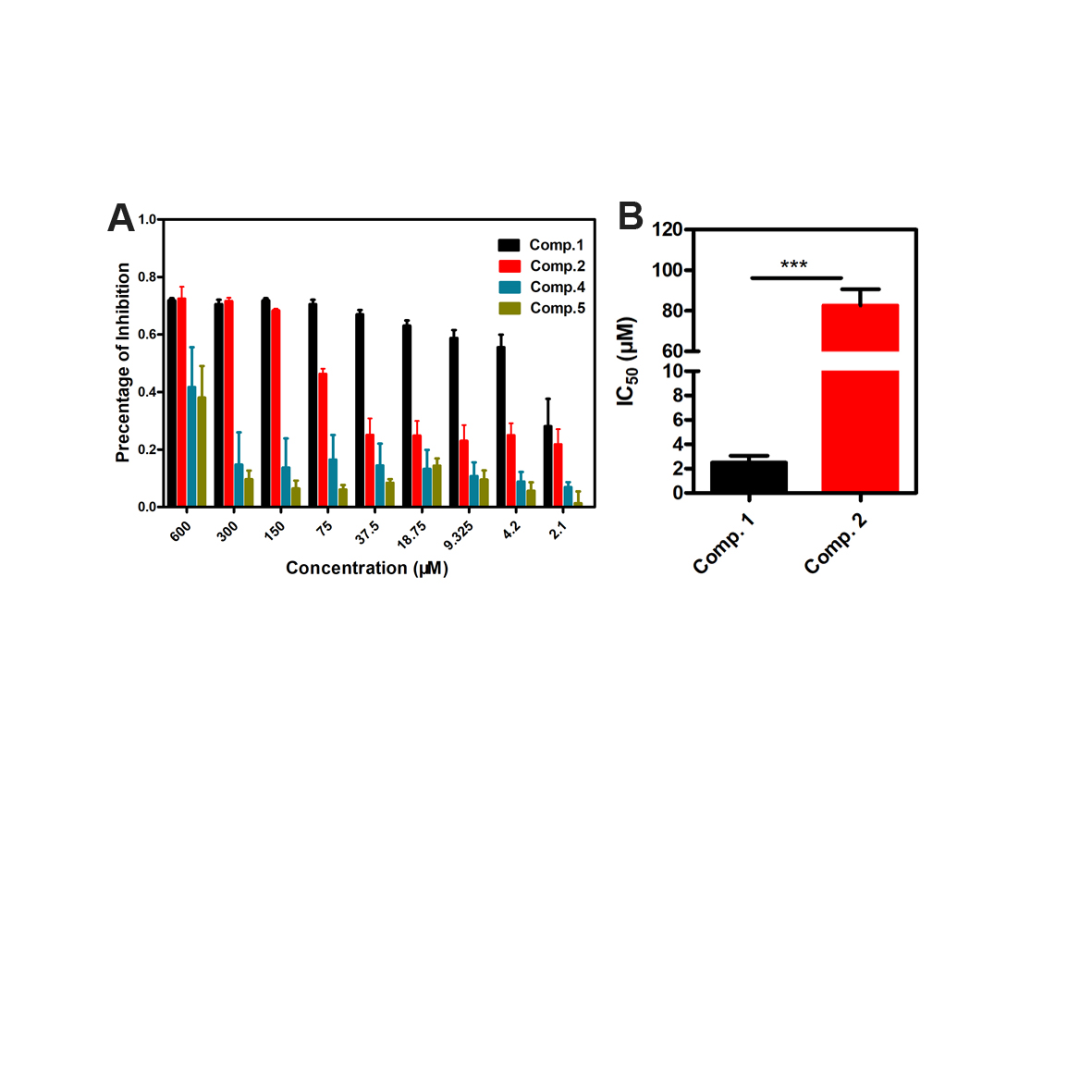


***Figure S31.*** A) Percentage of inhibition for A549 cells incubated with ***Comp.1***, ***Comp. 2***, ***Comp. 4*** and ***Comp. 5*** respectively. *Mean ± SEM, n=3.*


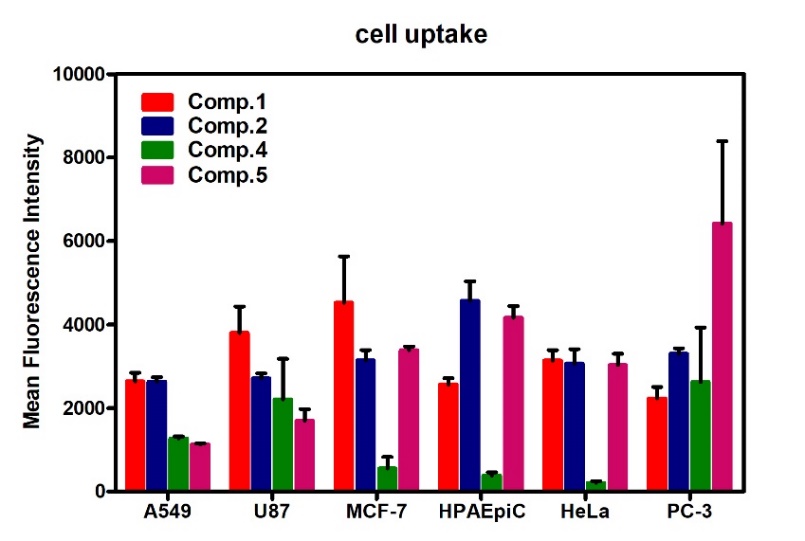


***Figure S32.*** Cellular uptake of compounds in different cell lines. *Mean ± SEM, n=3.*


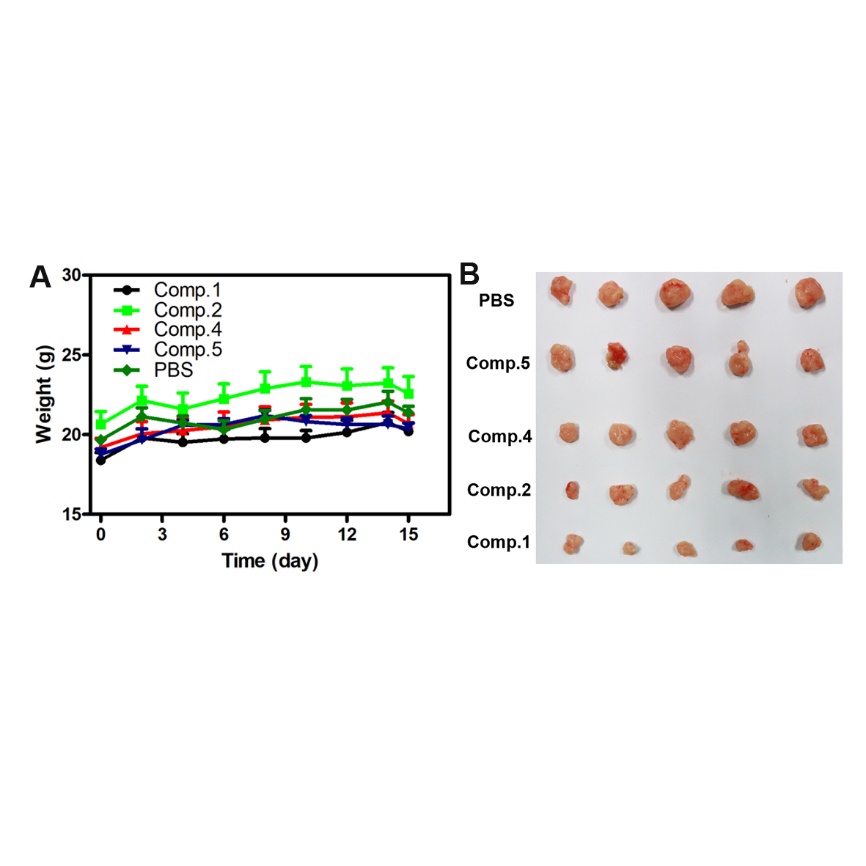


***Figure S33.*** (A) Weight change of mice treaded by different compound and PBS. *Mean ± SEM, n=3.*
